# Origin and Development of the Adipose Tissue, a Key Organ in Physiology and Disease

**DOI:** 10.3389/fcell.2021.786129

**Published:** 2021-12-21

**Authors:** Esmeralda Parra-Peralbo, Ana Talamillo, Rosa Barrio

**Affiliations:** ^1^ Faculty of Biomedical and Health Sciences, European University, Villaviciosa de Odón, Spain; ^2^ Center for Cooperative Research in Biosciences (CIC BioGUNE), Basque Research and Technology Alliance (BRTA), Derio, Spain

**Keywords:** WAT, BAT, beige adipocytes, adipose tissue, adipose stem cells, drosophila, fat body development, adepithelial cells

## Abstract

Adipose tissue is a dynamic organ, well known for its function in energy storage and mobilization according to nutrient availability and body needs, in charge of keeping the energetic balance of the organism. During the last decades, adipose tissue has emerged as the largest endocrine organ in the human body, being able to secrete hormones as well as inflammatory molecules and having an important impact in multiple processes such as adipogenesis, metabolism and chronic inflammation. However, the cellular progenitors, development, homeostasis and metabolism of the different types of adipose tissue are not fully known. During the last decade, *Drosophila melanogaster* has demonstrated to be an excellent model to tackle some of the open questions in the field of metabolism and development of endocrine/metabolic organs. Discoveries ranged from new hormones regulating obesity to subcellular mechanisms that regulate lipogenesis and lipolysis. Here, we review the available evidences on the development, types and functions of adipose tissue in *Drosophila* and identify some gaps for future research. This may help to understand the cellular and molecular mechanism underlying the pathophysiology of this fascinating key tissue, contributing to establish this organ as a therapeutic target.

## Introduction

In this manuscript we review the past and recent literature on the origin, development, types and function of mammalian adipose tissue and put it in relation to physiological and disease conditions such as obesity, diabetes, lipodystrophies or cancer-associated cachexia. We identify the gaps that need to be addressed regarding the origin and development of this tissue and propose *Drosophila* as a suitable model organism to explore those open questions. We review the existing evidences on the origin, development and function of the adipose tissue (AT) of this organism, making a clear distinction between the embryonic and larval stages and the adulthood, when the developmental programmes are finished. As we considered there are two different scenarios where play different actors and also where same actors could play different roles. We also review the available studies in *Drosophila* on the above-mentioned metabolic diseases.

## Mammalian Adipose Tissue and Associated Diseases

The lifestyle of developed countries, where population have an easy access to high caloric food and decreased physical exercise, has played important roles into the rise of obesity and Type 2 Diabetes Mellitus (T2DM) to the category of pandemics (Doria et al., 2008; Guilherme et al., 2008; Saltiel, 2012). Global prevalence of overweight and obesity combined has risen by 27.5% for adults and 47.1% for children between 1980 and 2013 (Ng et al., 2014). Furthermore, excess body weight is one of the major risk factors contributing to the global incidence of disease worldwide. According to the International Diabetes Federation (IDF), more than 371 million people across the globe have diabetes and this number is predicted to rise to over 550 million by 2030. Adipose tissue (AT) plays also important roles in other diseases. For instance, lipid storages at AT are susceptible to be wasted by tumour-secreted molecules, a fact known as cancer-associated cachexia (Rydén and Arner, 2007; Tsoli et al., 2016). This process is featured by increased systemic inflammation, general metabolic dysfunction, and elevated resting energy expenditure (Fearon et al., 2011). Cachexia affects 50**–**80% of cancer patients and accounts for up to 20% of cancer deaths. It is estimated that death normally ensues when weight loss exceeds 30**–**40% (Arthur et al., 2014). In this scenario, the understanding of adipocytes’ development will improve our knowledge on metabolic diseases such as obesity and T2DM, as well as on anomalous metabolic states that lead to chronic inflammation. (Stephens, 2012; Wu et al., 2013; Hotamisligil, 2017). Understanding in deep the biology of the AT will allow the development of potential therapies targeting thermogenesis as a means of increasing energy expenditure.

### The Adipose Tissue as a Regulator of Energy Homeostasis in Mammals

Energy is fundamental for life. Therefore, storage and homeostasis of energy are key processes for any organism. In the mammalian body, including that of humans, the energy that is neither consumed nor converted into glycogen is stored in form of neutral lipids in the adipocytes of the AT, more specifically in the lipid droplets (LDs). The LDs are key organelles controlling fat storage and mobilization (Olofsson et al., 2008; Beller et al., 2010). They consist of a core of neutral lipids (triglyceride and cholesterol esters) surrounded by a monolayer of phospholipid and cholesterol in which several proteins are embedded (Thiam and Beller, 2017).

Importantly, the AT is a plastic organ able to adapt to different physiological circumstances to ensure energy distribution among the different needs: metabolism, thermogenesis and lactation (Cinti, 2018). AT can grow by either increasing the number of adipocytes, which depends on adipocyte stem cells (ASC) or increasing the LDs size. In fat oxidizing tissues, LDs expansion is supported by specific mitochondria, known as peridroplet mitochondria. Those remain bound to the LD even after the homogenization the tissue and show specific features such as enhanced bioenergetic capacity, reduced β-oxidation capacity, supported LD expansion by providing ATP for triacylglycerides (TAG) synthesis, and maintenance of a distinct protein composition due to low fusion-fission dynamics (Benador et al., 2018).

In mammals there are different types of ATs: white AT (WAT), designed for energy storage, and brown AT (BAT), intended to dissipate energy and generate heat (Berry et al., 2013; Gupta, 2014). WAT is classified in subcutaneous or visceral WAT, depending on its anatomical location (Cleal et al., 2017). The two types of WATs have distinct developmental timing, microscopic appearance, molecular signature and certainly biological function. Subcutaneous WAT may protect against certain aspects of metabolic dysfunction (Snijder et al., 2003a; Snijder et al., 2003b). Visceral WAT is associated with metabolic complications and appears to increase the risk of T2DM, hyperlipidemia and cardiovascular disease (Grauer et al., 1984). Increasing number of evidences converge on to the idea that within mammalian bodies there are different AT depots, which present a vast heterogeneity among them, and contain the ASC that support their homeostasis (Cleal et al., 2017).

Until recently, the functions of the BAT were associated to the neonatal period (Novak et al., 1971) and the scientific community thought that this type of fat was not present during the adulthood. A bit more a decade ago, BAT was identified in adult humans and it was found to be reduced, on mass and activity, in obese and diabetic patients (Cypess et al., 2009; Cypess et al., 2013; Lidell et al., 2013; Alcalá et al., 2019). This finding pointed out to the idea of enhancement of BAT preadipocyte differentiation and proliferation as a therapeutic strategy to fight obesity (Alcalá et al., 2019). Later evidences converged on to the idea that adult human BAT shares molecular characteristics with murine “beige” cells rather than classical brown cells (Wu et al., 2012; Cannon et al., 2020).

“Beige” or “brite” adipocytes, also known as Beige Adipose Tissue (BeAT), are energy-burning adipocytes, with “brown-like’ features, such as increased mitochondrial content, multilocular storing of LDs, and the ability to burn off lipids as heat (Ikeda et al., 2018). BeAT is found in different spots of the adult body within the WAT (Cypess et al., 2009; Cypess et al., 2013; Lidell et al., 2013; Wu et al., 2012; Whittle et al., 2011). Although beige adipocytes share some markers with brown adipocytes, they also show specific markers different from those of both brown and white adipocytes (Table 1 **)** (Sharp et al., 2012; Waldén et al., 2012; Ussar et al., 2014)**.**

**TABLE 1:** Factors expressed in differentiated adipocytes.

Marker	Adipocyte type
LEP, ASC1	White
TEMEM26, HOXC9, TBX1	Beige
UCP1, PRDM16, P2RX5	Beige and brown
ZIC1, LHX8	Brown
ADIPQ	White, beige and brown

Abbreviations: ADIPQ, adiponectin; ASC1, asc-type amino acid transporter 1; HOXC9, homeobox C9; LEP, leptine; LHX8, LIM homeobox 8; P2RX5, purinergic Receptor P2X, ligand-gated ion channel 5; PRDM16, PR domain containing 16; TEMEM26, transmembrane protein 26; TBX1, t-box 1; UCP1, uncoupling protein 1; ZIC1, zinc finger protein of the cerebellum 1.

In spite of their distinct functions, WAT and “Beige AT” (BeAT) share the ability for reciprocal, reversible transdifferentiation to tackle special physiologic needs. Thus, chronic need for thermogenesis induces browning and chronic positive energy balance induces whitening (Cohen and Spiegelman, 2016; Cinti, 2018). Different signals, after birth and adult state, will determine BeAT differentiation. While Platelet activating factor and Interleukin 6 (IL-6) are determining factors in BeAT development after birth, β-adrenergic stimulation and IL-4 are active during adulthood (Chung et al., 2017; Finlin et al., 2017; Babaei et al., 2018; Yu et al., 2019; Hoang et al., 2021). Interestingly, a switch from BeAT to WAT underlies cancer-associated cachexia, triggered by the parathyroid-hormone-related protein (Kir et al., 2014; Petruzzelli and Wagner, 2016).

### The Adipose Tissue in Signalling and Inflammation in Mammals

AT is a large endocrine organ, insulin sensitive, that secretes around 600 different adipokines, the hormones that act on distant organs **(** Table 2) (Lehr et al., 2012a; Cinti, 2018) as well as a vast diversity of other signalling molecules, such as metabolites, lipids, non-coding RNAs or extracellular vesicles (Funcke and Scherer, 2019). Leptin and adiponectin are well known hormones secreted by adipocytes (Cinti, 2018). Leptin inhibits appetite, stimulate thermogenesis, enhance fatty acid oxidation, decrease glucose, and reduce body weight and fat. Adiponectin mediates the insulin-sensitizing effect (Yadav et al., 2013). Omentin, secreted by non-adipocyte cells in the AT, acts as insulin-sensitizing factor and it has been reported to have anti-inflammatory, anti-atherogenic and anti-cardiovascular disease properties (Tan et al., 2010). Dipeptidyl peptidase IV, secreted by visceral white adipocytes from obese individuals, seems to be associated to insulin-resistance development (Lamers et al., 2011; Lehr et al., 2012b).

**TABLE 2:** Mammalian AT secreted molecules.

	—	—
Adipokines	Leptin	LEP
	Adiponectin^a^	ADIPOQ
	Resistin	RETN
	Fibrillin 1 (aprosin)^a^	FBN1
	Serpin family E member 1 (plasminogen activator inhibitor)	SERPINE1 (PAI-1)
	Apelin^a^	APLN
	Intelectin 1 (omentin)	ITLN1
	Retinol-binding protein 4	RPB4
	Nicotinamide phosphoribosyltransferase (visfatin)	NAMPT
	Nucleobindin 2 (nesfatin 1)	NUCB2
	Dipeptidyl peptidase IV	DPP-4
	Endocannabinoids	
Alternate complement system	Complement C3	C3
	Complement factor B	CFB
	Complement factor D (adipsin)^a^	CFD
Growth factors	Fibroblast growth factor-21	FGF21
	Bone morphogenetic protein	BMP
Vasculotrophic factors	Vascular endothelial growth factor	VEGFA
	NO	
	CO	
	Angiotensin II	AGT
Neurotrophic factors	Nerve growth factor	NGF
	Semaphorin 3 and 6	SEMA3A/SEMA6A
	Neuregulin 4	NRG4
Inflammatory cytokines	Tumor necrosis factor α	TNFα
	Interleukin 6	IL6
	Interleukin 33	IL33
	Interleukin 1B	IL1B
	C-C Motif chemokine ligand 5	CCL5 (RANTES)
	Interleukin 8	IL8
	C-X-C Motif chemokine ligand 12 (stromal cell-derived factor 1)	CXCL12 (SDF1)
	Macrophage migration inhibitory factor	MIF
	C-C Motif chemokine ligand 2 (Monocyte chemoattractant protein-1)	CCL2 (MCP1)
Lipid metabolism	Lipoprotein lipase	LPL
	Cholesteryl ester transfer protein	CETP
BAT-adipokines	Peptidase M20 domain containing 1	PM20D1

a Secreted also by skeletal muscles. In parenthesis, other used names for the same factor. NO, nitric oxide; CO, carbon monoxide.

AT is not only composed by adipocytes but also comprises as well other cell types such as preadipocytes, fibroblasts, stromal cells, T-cells, granulocytes, macrophages and monocytes (Hotamisligil, 2017). During the last years, several adipokines and cytokines secreted by AT and also other molecules of signalling pathways linking AT metabolism and immune system, have been identified. For example resistin, a hormone secreted by macrophages M1, pro-inflammatory phenotype, that infiltrate obese adipoctyes, produces a notable effect on systemic metabolism by acting as a crosstalk between obesity-inflammation and metabolic diseases (Schwartz and Lazar, 2011). Likewise, the dysfunction of AT is associated with the secretion of multiple molecules that mediate the inflammatory response. In fact, stressed adipocytes from obese ATs activate the inflammasome system, which could induce a chronic low-grade inflammation. Inflammation appears in other tissues besides AT, including brain, liver, airways and pancreatic islets. This inflammatory state is known as “metaflammation” because it contributes to several immunometabolic diseases, including T2DM, cardiovascular disease, asthma, neurodegenerative disease, cancer and lipodystrophies (Hotamisligil, 2017; Cinti, 2018).

### The Origin and Development of the Mammalian Adipose Tissue

A great number of studies, most of them carried out *in vitro*, shed light on the factors involved in differentiation of white, brown and beige adipocytes, such as peroxisome proliferator-activated receptor gamma (PPARγ), CCAAT/enhancer binding proteins (C/EPB), krupper-like factors (KLFs), peroxisome proliferator-activated receptor gamma coactivator-1 alpha (PGC1a) or nuclear factor I A (NFIA) (reviewed at (Hiraike et al., 2017; Cinti, 2018; Hiraike et al., 2020). However, the initial commitment of mesenchymal progenitors to the adipocyte lineage remains less explored (Billon et al., 2007).

A comprehensive understanding of the origin of white/brown/beige adipocytes differentiation is of upmost interest given the potential to induce browning AT in obese patients (Cypess and Kahn, 2010). It is accepted that animals with more BAT are more resistant not only to obesity but also to T2DM (Kopecký et al., 1996; Collins et al., 1997; Guerra et al., 1998; Almind et al., 2007). Conversely, animals without functional BAT are prone to obesity and T2DM (Lowell et al., 1993; Bachman et al., 2002; Feldmann et al., 2009). Attempts to block lipid storage or inhibiting WAT development failed, as several studies have shown that this strategy drives fat accumulation in organs not specialized for fat storage. This condition is known as “ectopic lipid” and has been associated with insulin resistance and the development of T2DM (Gastaldelli, 2011).

BAT, WAT and BeAT can develop from both neural crest and mesoderm, specifically BAT develops from paraxial mesoderm and WAT and Beige AT does from lateral plate mesoderm (Billon et al., 2007). Periaortic arch adipose tissue (PAAT), which is composed of both BAT and WAT, is derived from multiple cell lines, being neural crest cells the main contributors (Fu et al., 2019). Furthermore, brown adipocytes could arise from Myogenic Factor 5 (MYF5)-expressing myogenic precursor cells by the action of PRDM16 (PRD1-BF1-RIZ1 homologous domain containing 16). PRDM16 controls a bidirectional cell fate switch between skeletal myoblasts and BeAT cells and it is also responsible for beige cells found within WAT depots (Seale et al., 2008; Enerbäck, 2009; Lshibashi and Seale, 2010; Petrovic et al., 2010; Seale et al., 2011). PRDM16 gene expression is downregulated by miR-149-3p during fasting conditions allowing the switch from subcutaneous to visceral AT (Ding et al., 2016).

The specific origin of the beige adipocytes remains to be clarified. Some evidences support the idea that they arise from unique precursor cells (Wu et al., 2012), but others suggest that they may arise from white adipocytes in a process referred to as transdifferentiation (Cinti, 2012; Cinti, 2018).

Moreover, developed spots of both BAT and WAT present a remarkable adipocytes’ heterogeneity. Low- and high-thermogenic brown adipocytes with distinct features and functions coexist in BAT. Low-thermogenic brown adipocytes, unlike the high-thermogenic ones, show low *Ucp1* and *Adipoq* expression, larger lipid droplets, lower mitochondrial content and are functionally specialized in fatty acid uptake (Song et al., 2020). Similarly, functional heterogeneity is found when comparing subcutaneous and omental preadipocytes, which show distinct capacities for replication, adipogenesis and apoptosis (Tchkonia et al., 2005).

To study in deep all aspects of adipocyte biology is required to know the molecular properties of adipose precursor cells and the ontogeny of fat cells *in vivo* (Gupta, 2014). Understanding the origin and differentiation of the different types of adipocytes might pave the way for future therapies for obesity, T2DM, cancer-associate cachexia and immunometabolic diseases (Stephens, 2012; Jung et al., 2019).

## Open Questions on the Origin and Function of the Adipose Tissue

One of the most important open questions is the origin of the AT: which factor(s) are required for the specification of the AT primordium, how proliferation of ASC is regulated and how ASC differentiate into adipocytes. The identification and characterization of the ASC population is fundamental to understand AT development, formation and maintenance.

Equally important is to study how the ASC contribute to the homeostasis and maintenance of the AT under both normal energy intake and excess nutrient load. In the same way, it would be critical to know the growth factors and developmental signalling pathways altering ASC behaviour and adipocyte formation. Furthermore, although some factors determining fat cells fate has been described, most of these studies have been carried out *in vitro* and their role *in vivo* has not been explored (Berry et al., 2013; Gupta, 2014; Jung et al., 2019).

Last but not least, the growing number of functions in which the mammalian AT is involved require further studies, being the use of model systems an important tool to be exploited (Hotamisligil, 2017).

## *Drosophila* as a Model to Study Adipose Tissue

**FIGURE 1:**
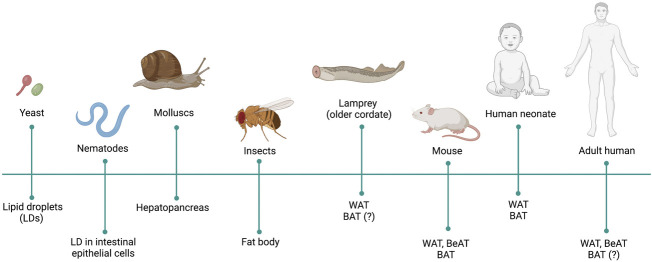
Evolution of the adipose tissue (AT). Presence of fat cells, fat organs and proper AT is indicated in different phyla and species throughout evolution.

The ability to store nutrients, mainly in form of TAG, is conserved from yeast to human (Kadereit et al., 2008) (Figure 1). Even though a better knowledge on AT from an evolutionary perspective could improve our understanding on the development, function and dysfunction of this organ, this theme remain as a neglected enigma (Ottaviani et al., 2011). TAG synthesis and lipolysis related genes are already present in unicellular organisms such as *Saccharomyces cerevisiae* or *Candida parapsilosis* (Zweytick et al., 2000; Neugnot et al., 2002; Daum et al., 2007). *Caenorhabiditis elegans* also stores TAG in form of LDs in the intestinal epithelial cells (Watts, 2009).

The ability to, not only store fat, but also to secrete endocrine factors is already present in molluscs. *Haliotis fulgens* or *Helix aspersa* store TAG in the midintestinal gland known as hepatopancreas. This also secretes a glucose lowering level hormone, Phe-Met-Arg-Phe-amide (FMRFa), belonging to the evolutionarily conserved RF-amide neuropeptide family (Rőszer and Kiss-Tóth, 2014). Neuropeptide FF (NPFF) an anorexigenic peptide, also member of this family, is critical to keep a basal NPY gene expression at arcuate nucleus and promote diet-induced thermogenesis, coupling energy homeostasis with energy partitioning to AT and bone tissue (Zhang et al., 2018). Moreover, NPFF is able to promote macrophage M2, anti-inflammatory phenotype, activation and increase the proliferation of murine and human adipose tissue macrophages (Waqas et al., 2017). Latter in evolution, the AT, in addition to serve as a fat storage and endocrine tissue, is also involved in immunity, as is the case of insects. The lipid storage tissue in insects is known as fat body (FB), which is an endocrine-secreting organ involved in nutrient sensing, development, metabolism, immunity and reproduction (Doane, 1960; Dean et al., 1985; Charroux and Royet, 2010).

WAT is present in most of vertebrate taxa, including fish, amphibian, reptiles and mammals (Gesta et al., 2007; Todorčević et al., 2009; Imrie and Sadler, 2010). Lampreys, at the base of vertebrates’ evolution, present fat cells with similar morphological characteristic to white and brown adipocytes (Müller, 1968), which is in conflict with the generally accepted idea about BAT is not present in cold-blooded vertebrates.

BAT is larger in small mammals, such as mouse, than in human. A recent debate points out to mice BAT, rather than the human adult BAT, as a classic defined BAT and, therefore, considers mouse the best model to study the development of this tissue (Cannon et al., 2020).

However, considering the complexity of the AT in mammals, a simpler model than mouse is required to study the development and determination of this tissue. A model organism that allows performing genetics analysis *in vivo* would be suitable to address those open questions. In this regard, *Drosophila* represents a good model for the study of AT based on its genetics accessibility, the lower complexity of the AT and the functional conservation of this tissue along evolution.

*Drosophila melanogaster* has been used for more than 100 years to study conserved biological processes and decipher the molecular and genetic basis of multicellular organisms, as well as a vast number of human diseases (Yamaguchi and Yoshida, 2018). Several studies have established *Drosophila* as a model to study obesity and metabolic diseases [reviewed at (Musselman and Kühnlein, 2018)]. Importantly, molecules and signalling pathways involved in the regulation of metabolism and physiology of the AT in mammals are conserved in *Drosophila*. For instance, *Drosophila* insulin/insulin like growth factor signalling (IIS) acts as a conserved satiety pathway promoting glucose uptake by peripheral tissues (Saltiel and Kahn, 2001) and sustaining sugar and lipid anabolic processes (Kim and Rulifson, 2004; Buch et al., 2008). Glucagon-like peptide adipokinetic hormone (Akh) signalling, conversely, is activated in response to reduced nutrient availability and promotes mobilization of energy reserves (Kim and Rulifson, 2004; Lee and Park, 2004; Bharucha et al., 2008). Short neuropeptide F (sNPF) is a functional homolog of mammalian orexigenic Neuropeptide Y (Nässel and Wegener, 2011), and its overexpression in sNPF-producing neurons causes hyperphagia and body fat accumulation in flies (Baumbach et al., 2014). Conversely, downregulation of this gene in sNPF-positive neurons reduces food intake (Lee et al., 2004). More recently, *Drosophila* has been demonstrated to be a good model to study T2DM. Flies fed on high sugar diet (HSD) develop diabetes showing increased levels of glucose in their hemolymph (blood-like system), insulin resistance and heart dysfunction (Palanker Musselman et al., 2011; Pasco and Léopold, 2012; Na et al., 2013). *Drosophila* has also been used as a model to identify new regulators of mammalian glucose metabolism (Ugrankar et al., 2015) and has made important contributions to understand the main components of signalling pathways involved in tumour development, including the cancer associated cachexia (Figueroa-Clarevega and Bilder, 2015; Enomoto et al., 2018; Saavedra and Perrimon, 2019).

**FIGURE 2:**
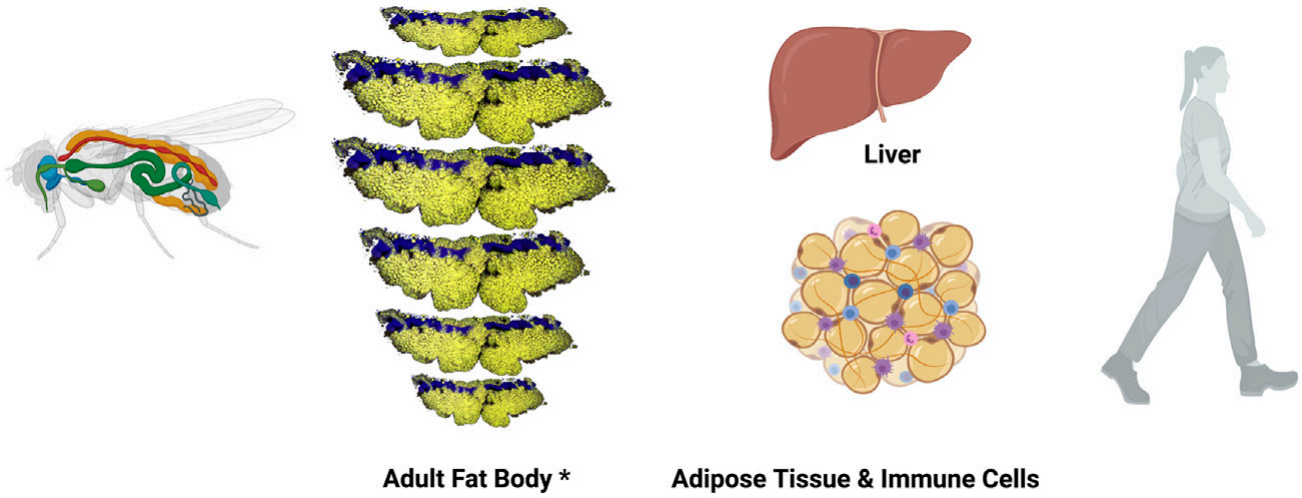
*Drosophila melanogaster* fat body and mammalian functional orthologous organs. Left, schematic representation of *Drosophila melanogaster* adult animal. Fat body is in orange, heat in red and digestive system in green. *Adult *Drosophila* FB is represented here using a composition made by repeating a confocal image of a single panicle of one the dorsal abdominal segments from an adult female fly. Confocal image shows adipocytes in yellow (Nile Red staining) and oenocyte nuclei in blue (DAPI staining). Right, illustrations show mammalian liver and adipose tissue (cells in orange) infiltrated with immune cells (blue and purple cells).

Studies to fully understand the regulation of lipid metabolism in *Drosophila* are ongoing. It is well known that lipids are taken by adipocytes from the hemolymph, and are esterified and stored as TAGs and cholesterol esters. Moreover, the *Drosophila* FB, functionally equivalent to mammalian AT and liver, carries out glycolysis and lipogenesis using carbohydrates (Figure 2) [reviewed at (Arrese and Soulages, 2010)]. Also, cellular lipid uptake as well as lipid transport and lipoprotein metabolism has been well studied in *Drosophila* (Parra-Peralbo and Culi, 2011; Palm et al., 2012; Rodríguez-Vázquez et al., 2015; Yin et al., 2021). Furthermore, fly mutants in Lipin, a phosphatidate phosphatase required for normal insulin pathway signalling that plays a central role in FB function and energy metabolism, Seipin, a transmembrane protein with roles in ER calcium homeostasis and lipid storage, or Sik3 (Salt-inducible kinase 3), a kinase involved in lipid catabolism by regulating *bmm* gene expression show reduced lipid content and lipodystrophy (Li et al., 2019). All together make this model organism suitable to study different types of lipodystrophies.

As Edward O. Wilson said in “Letters to a young Scientist” for, each biological question there is a suitable system for discovering the answer (Wilson, 2013). In that case, we, as Azeez and collaborators, think that *Drosophila melanogaster* is a suitable system to identify the primordium of AT and the population of ASCs in adults, as well as to characterize the adult AT in order to understand the adipocyte biology (Azeez et al., 2014). As a proof of principle, Pospisilik *et al* (Pospisilik et al., 2010) found Hedgehog as a determinant of Brown versus White adipose cell fate, using *Drosophila* as a model system. In addition, a well-established cell linage tracing system, G-TRACE, has been a key tool for exploring origin, development and differentiation of tissues in *Drosophila* (Evans et al., 2009), only very recently available in mammalian model systems (Berry et al., 2013; Jung et al., 2019).

## Origin and Development of Embryonic Fat Body Precursors

The *Drosophila* FB arises from the embryonic mesoderm (Hartenstein and Jan 1992). At stage 11, the progenitor fat cells arise from nine bilateral clusters of cells in the inner mesodermal layer that span the parasegments 4 through 12 and the mesoderm separates in the splanchnopleure and somatopleure. The somatopleure will give rise to the FB, somatic musculature and other cell types (Campos-Ortega and Hartenstein, 1985). FB cells’ lineage can be traced by analyzing the expression patterns of the genes *Alcohol dehydrogenase* (*Adh*), *Collagen type IV alpha 1* (*Col4a1*), the steroid hormone receptor *seven up* (*svp*) and *serpent* (*srp*), as well as the enhancer-trap line *29D* that exhibits an expression pattern restricted to developing embryonic fat cells (Hoshizaki et al., 1994) **(** Tables 3, 4 **)**. This enhancer-trap line allowed tracing the fat-cell lineage to nine bilateral cluster of cells within the emerging mesoderm, representing the progenitor fat cells. The *svp*-positive cells at stage 12 identified early precursor fat cells, and the expression of *Adh* and *Col4a1* was used to identify the terminal fat cell differentiation at stage 15. By late stage 15/16 embryo, mature fat cells coalesce into a single cell thick FB layer throughout the abdomen and form three domains: the lateral FB, the dorsal fat cell projection, and the ventral collar (Miller et al., 2002). Finally, the expression of the GATA-like transcription factor Srp is a marker for the early stages of fat cell development (Sam et al., 1996). Other enhancer traps-lines that drive expression in fat cells at larval and adult stages are *3-76a*, *X8-157a,* and *l(3)2E2.* Interestingly, *l(3)2E2* regulates *svp* gene expression (Hoshizaki et al., 1995). Therefore, *svp* and the gene(s) near to the enhancer trap *29D* were suggested to be key factors for determination and differentiation of embryonic FB (Tables 3, 4) (Hoshizaki et al., 1994).

**TABLE 3:** Factors and signalling pathways playing a role in fat body development or function.

Factor/pathway	Abbreviation	Human homolog	Stage	Function	References
Adipokinetic hormone (Akh)/Akh receptor signalling	Akh/AkhR	Functional homolog to glucagon	L, A	Carbohydrate and lipid mobilization	Fu et al., (2019), Seale et al., (2008), Enerbäck (2009), Daum et al. (2007), TM. (1978), Butterworth et al., (1988), Britton and Edgar. (1998), Guarner et al., (2017), Britton et al. (2002), Hoshizaki. (1994)
Alcohol dehydrogenase	Adh	15-hydroxyprostaglandin dehydrogenase	E	Fat metabolism	Todorčević et al. (2009)
Bigmax	Bigmax	Max-like protein X	L	Sugar sensing and lipogenesis	Arrese and Soulages, (2010)
Brummer lipase	Bmm	Adipose triglyceride lipase, ATGL	L, A	Lipolysis independent of Akh	Yamada et al. (2018)
Cabut	Cbt	Kruppel-like factors 10 and 11	L	Transcriptional repression upon sugar sensing	Arrese and Soulages, (2010)
cAMP-responsive element binding protein B	dCREB2	CREB/CREM	A	Akh target. TAG storage modulation	(Seale et al., 2008; Ren et al., 2015)
CCHamide-2^a^	CCHa2	Neuropeptide	L	Ilp2 and 5 expression and secretion	Ugrankar et al., (2019), Ugrankar et al. (2011)
Dawdle^a^	Daw	Activin	L	DILPs secretion, inhibition of carbohydrase and lipase at intestine	Bi et al. (2012), Beller et al. (2006)
Dorsal	Dl	RELA proto-oncogene	L	Toll target, induced by fungi and Gram-positive bacteria	Grönke et al. (2010)
Dorsal-related immunity factor	Dif	RELA proto-oncogene	L	Toll target, induced by fungi and Gram-positive bacteria	Grönke et al. (2010)
DP Transcription Factor	DP	Transcription Factor Dp-1, TFDP1	L	Endoreplication	Baumbach et al. (2014)
*Drosophila* insulin/insulin like growth factor (IGF) signalling	ISS	Insulin like signalling	L	Coordination of nutritional status, endoreplicating tissue metabolism and growth. Determination of final body size. Inhibition of immune gene expression	Feldmann et al., (2009), Gastaldelli, (2011), Baumbach et al. (2014), Fu et al., (2019), Lee et al., (2004), TM. (1978), Palanker et al., (2009), Reis et al., (2010), Bartok et al. (2015), Palu and Thummel (2016)
E2F Transcription Factor1, 2	E2f1, E2f2	E2F Transcription Factor 1-6	L	Endoreplication	Baumbach et al. (2014)
Ecdysone signalling	Ec	NF	L	Antagonist to ISS, systemic growth inhibition	Walther and Farese, (2012)
Eiger^a^	Egr	Tumor necrosis factor alpha, TNFalpha	L	Activation of JNK-dependent inhibition of Ilps production	Pancreatic Hormones (1990)
Endoplasmic reticulum degradation enhancing α-mannosidase-like protein 1	Edem1	ER degradation enhancing alpha-mannosidase like protein 2	L	Systemic insulin signaling maintenance	Isabel et al. (2005)
Extracellularly regulated kinase 7	Erk7	Mitogen-activated protein kinase 15	L	Growth, lipid storage and adaptation to nutrient shortage	Riechmann and Rehorn, (1998)
Forkhead box, sub-group O	Foxo	FOXO3	L, A	Inhibition of Daw expression. Increased lifespan	Werthebach et al. (2019)
Glass bottom boat	Gbb	Bone morphogenetic protein 7	L	FB development and metabolic homeostasis	Moore et al. (1998)
Growth blocking peptide 1^a^	GBP1	Epidermal growth factors, EGF	L	Induction of Ilp secretion	Hasygar et al. (2021)
Growth blocking peptide 2^a^	GBP2	Epidermal growth factors, EGF	L	Induction of Ilp secretion	Hasygar et al. (2021)
Hepatocyte nuclear factor 4	Hnf4	Hepatocyte nuclear factor 4 gamma	L	Carbohydrate metabolism	Palm et al. (2012), Rodríguez-Vázquez et al., (2015)
Histone deacetylase 4	HDAC4	HDAC	A	Akh target under short fasting condition. Lipolysis	Delanoue et al. (2016), Koyama and Mirth. (2016)
Imaginal morphogenesis protein-late 2^a^	ImpL2	Insulin-Like Growth Factor Binding Protein 7, IGFBP7	L, A	Binds DILPs extracellularly and inhibits ISS, tumour-mediated FB wasting	Kadereit et al., (2008), Schaffer et al., (1990)
Immune deficiency signalling	Imd	NF	L	Immunity, inhibition of growth, reduction of ISS/TOR signalling and TAG storage	Grönke et al., (2010), Post et al., (2018)
Insulin like peptide 2	Ilp2	Insulin	L	Regulate glycogen synthesis	Fu et al. (2019)
Insulin like peptide 3	Ilp3	Insulin	L	Synthesis and release of trehalose into hemolymph	Butterworth et al. (1988)
Insulin like peptide 5	Ilp5	Insulin	L	Regulate glycogen synthesis	Fu et al. (2019)
Insulin like peptide 6^a^	Ilp6	Insulin	L	Toll pathway target, repression of DILP2, lifespan extension	Zimmermann et al., (2004), Heine et al., (2018), DiAngelo and Birnbaum, (2009)
Insulin like peptide 7	Ilp7	Insulin	L	Regulation of TAG synthesis	Palu and Thummel, (2016)
Kruppel	Kr	BCL6 transcription repressor	L	Fat determination/differentiation (?)	Todorčević et al., (2009), Palanker Musselman et al., (2011)
Lipid storage droplet-1	Lsd-1	Perilipin 2	L	Lipolysis	Evans et al. (2009), Campos-Ortega and Hartenstein. (1985)
Lipid storage droplet-2	Lsd-2	Perilipin 2	L	Involved in TAG storage	(Hartenstein and Jan 1992, Campos-Ortega and Hartenstein, (1985)
Lipin	Lpin	Lipin 3	L	FB development and TAG storage	Hoshizaki et al. (1995)
Liver kinase B1	Lkb1	Liver kinase B1	A	Akh/AkhR signalling target under short fasting condition. Lipolysis	Delanoue et al., (2016), Koyama and Mirth, (2016)
mir-8 stem loop	miR-8	microRNA 200a	L	Ec signalling target, growth regulation	Schaffer et al., (1990), Hughson et al. (2021), Young and Zechner. (2013)
Mondo	Mondo	MLX interacting protein	L	Sugar sensing and lipogenesis	Enomoto et al., (2018), Saavedra and Perrimon, (2019), Arrese and Soulages, (2010)
Myc	Myc	MYC proto-oncogene	L	Ec signalling target, control of glucose and lipid metabolism, Ilp2 secretion	Schaffer et al., (1990), Arrese et al., (2006), Gáliková et al., (2015)
NAD + dependentdeacetylase Sirtuin 1	Sirt1	Sirtuin 1	L	Inhibition of TAG storage	Yin et al. (2021)
NAD + dependent deacetylase Sirtuin 2	Sirt2	Sirtuin 2	L, A	Glucose homeostasis and peripheral insulin sensitivity. Increased lifespan	Rodríguez-Vázquez et al. (2015)
No child left behind	Nclb	PWP1 homolog	L	ERK7 target, growth-promoting downstream effector of mTOR	Riechmann and Rehorn, (1998)
PDGF- and VEGF-related factor 1	Pvf1	Platelet derived growth factor	A	Repression of lipid synthesis by activating TOR signaling at oenocytes at the end of AT development. Tumour-mediated FB wasting	Luong et al., (2006), Sousa-Nunes et al., (2011)
protein 53	p53	protein 53	L	Sensing nutrient stress and metabolic homeostasis, AMPK target	Grönke et al. (2003)
Relish	Rel	Nuclear factor kappa B subunit 1	L	Imd target, induced by Gram-negative bacteria	Grönke et al. (2010)
Salt-inducible kinase 3	Sik3	SIK family kinase 3	A	Akh/AkhR signalling target under short fasting conditions. Insulin target feeding conditions. Lipolysis	Delanoue et al., 2016, Koyama and Mirth, 2016)
Serpent	Srp	GATA binding protein 1	E	Fat determination/differentiation	Todorčević et al., (2009), Saltiel and Kahn, (2001), Buch et al., (2008), Musselman and Kühnlein, (2018)
Seven up	Svp	Nuclear receptor subfamily 2 group F member 2	E, L, A	Fat determination. Immunity and xenobiotic response	Todorčević et al. (2009)
Slimfast	Slif	Solute carrier family 7 member 1	L	Amino acid sensing	Cermelli et al. (2006)
Snazarus	Snz	Sorting nexin 25	L	Activation of TAG storage a t peripheral LD	Sam et al. (1996)
Stearoyl-CoA desaturase	Desat1	Stearoyl-CoA desaturase 5	L	Fatty acids and lipid biosynthesis	Arrese et al. (2006)
Store-operated calcium entry	SOCE	Store-operated calcium entry	A	Akh/AkhR signalling target. TAG storage modulation	Petrovic et al. (2010)
Stunted^a^	Sun	ATP synthase F1 subunit epsilon	L	TOR signalling target, Ilp secretion	Ballard et al. (2010)
Sturkopf	Sturkopf	Lipid droplet associated hydrolase	L	Endocrine physiology regulation (ISS and JH pathway)	Miller et al. (2002)
Sugarbabe	Sug	Gli-similar transcription factor	L	ERK7 target, lipogenic TF	Enomoto et al., (2018), Riechmann and Rehorn (1998)
Target Of Rapamycine signalling	TOR	mTOR signalling	L	Cellular nutrient sensing	Cermelli et al., (2006), Krahmer et al. (2013), Grönke et al. (2003), Ballard et al. (2010)
Telomere fusion	Tefu	ATM serine/threonine kinase	L	E2F/D*P* target, inhibition of DNA damage response	Lee et al. (2004)
Triglyceride Lipase	TGL	Lipase A, lysosomal acid type	L	Lsd-1 target. Lypolisis	Britton et al. (2002)
Toll signalling	Toll	Toll-like receptor family signalling	L	Immnunity, inhibition of growth, reduction of ISS signalling and TAG storage	Palanker et al., (2009), DiAngelo and Birnbaum, (2009), Okamoto et al. (2009), Slaidina et al. (2009), Grönke et al. (2010)
Type IV collagen	Col4a1	Collagen type IV alpha 1 chain	E	Fat metabolism	Todorčević et al. (2009)
Uncouple protein 4C	Ucp4C	Uncouple protein 1	A	Dissipation of energy in the mitochondria	Rajan and Perrimon, (2012), Ingaramo et al., (2020)
Unpaired 2^a^	Upd2	JAKSTAT ligand, functional homolog to leptin	L	p53 target, DILPs secretion	Teixeira et al., (2003), Grönke et al. (2003)

a FB-secreted factors. Abbreviation: NF, not found; E, embryo; L, larvae; A, adult.

**TABLE 4:** Enhancer trap lines.

Enhacer trap	Cells	Cytological location
29D	EFC	58DE
l (3)2E2	EFC, LFC, AFC	87B (*seven up*)
3-76a	EFC, LFC, AFC, ADEC	5CD
X8-157a	EFC, LFC, AFC, ADEC	19D
RD721	LFC, AFC	58C
RD1937	LFC, AFC	3CD
l (2)0734	LFC, AFC	Chr 2
l (2)895	LFC, AFC	60F (*kruppel*)
l (2)3552	LFC, AFC	Chr 2
l (2)10,435	LFC, AFC	Chr 2
l (3)4504	LFC, AFC	Chr 3
l (3)7842	LFC, AFC	Chr 3
S3358	LFC, AFC	26D
rP445	LFC, AFC	24A
AS3	LFC, AFC	25BC
RD1272	LFC, AFC	64B
RD61	AFC	54BC

Abbreviations: EFC, embryonic fat cells; L, larvae fat cells; A, adult fat cells; ADEC, adepithelial cells. In parenthesis, genes probably regulated by those enhancers.

The development of the FB requires the GATA-like transcription factor Srp, necessary and sufficient for the progression through the early stages and development of fat cells (Saltiel and Kahn, 2001; Buch et al., 2008; Musselman and Kühnlein, 2018). In fact, FB and gonads derive from mesoderm and abdA allows gonadal mesoderm to develop by repressing Srp function in this region (Moore et al., 1998).

## Origin and Development of the Larval Fat Body

The larval FB is a single cell layer that spreads along the larval body cavity, surrounding the gut and reproductive organs and being exposed to the hemolymph (TM. (1978); Dean et al., 1985). Larval FB contains 2200 cells, a number that remains constant throughout FB development. At larval stages, the FB growth is achieved by increasing cell size through endoreplication cycles, with successive rounds of DNA synthesis without mitosis (Butterworth et al., 1988; Britton and Edgar, 1998). Cell size changes are associated with the accumulation of LDs, glycogen deposits and protein granules. The endocycling progression in the FB cells requires the heteromeric transcription factor complex E2f1/E2f2/DP to repress *telomere fusion (tefu)* and suppress DNA damage responses (Guarner et al., 2017). In addition, endoreplication in the FB cells is tightly regulated in response to nutrition and depends on IIS (Britton et al., 2002).

Evidences suggest that the development of larval FB might require the expression of various unidentified genes, revealed by the expression of a number of enhancer traps (Table 4) including *3-76a*, *X8-157a, l(3)2E2*. Specifically, the last one regulates the gene expression of *svp*, suggesting that Svp activity might be involved in that process. *kruppel* (*kr*) expression is not detected in fat cells during embryogenesis, nor during the first- and second-instar stages. However, Kr is expressed in fat cells at the stage previous to metamorphosis and in adults **(** Table 4). It is possible that Kr serves as a transcriptional regulator in the FB in this last larval instar (Table 3) (Hoshizaki, 1994; Hoshizaki et al., 1994). According to that, it has been found that Kruppel-like factor 11 (KLF11) is a novel browning transcription factor in human adipocytes (Loft et al., 2015).

At the end of the larval development, the FB undergoes a remodelling process with massive autophagy that initiates the pupal transition. The larval FB decreases gradually throughout metamorphosis, and during the first 3 days of adulthood, until no more cells can be observed.

## Roles of the Larval Fat Body

**FIGURE 3:**
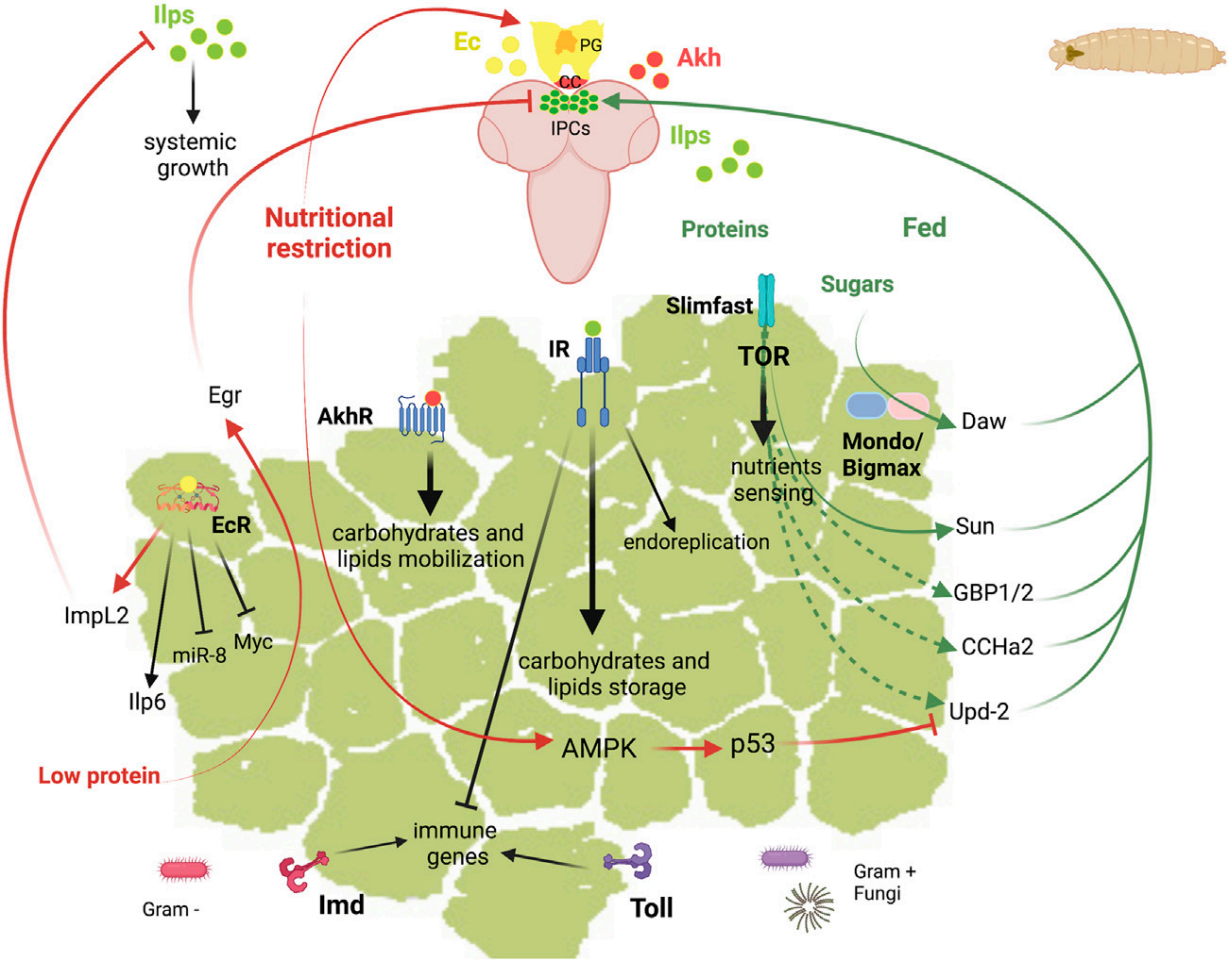
Schematic representation of the diverse functions of *Drosophila* fat body in larvae. Top, larvae brain represented in soft purple. Abbreviations: Akh Adipokinetic hormone; AkhR, Akh receptor; AMPK, AMP-activated protein kinase; CC, corpora cardiaca; CCHa2, CCHamide-2; Daw, Dawdle; Ec, ecdysone; EcR, ecdysone receptor; Egr, Eiger; GBP1/2, Growth blocking peptide 1/2; ImpL2, Imaginal morphogenesis protein-late 2; Imd, Immune deficiency; Ilps, insulin-like peptides; Ilp6, Insulin-like peptide 6; IPC, insulin producing cells; IR, insulin receptor; miR-8, mir-8 stem loop; PG, prothoracic gland; Sun, Stunted; Svp, Seven up; TOR, target of rapamycin; Upd2, Unpaired 2.

The *Drosophila* larval FB is involved in multiple functions that allow the coordination of the metabolic homeostasis. Larval FB extends as a longitudinal fat sheet at each larval body side. Salivary glands present also an associated-FB whose function is unknown. The most important functions of this tissue include the storage and release of energy, the nutrient sensing function, and the role in the systemic immunity (Figure 3 **)**. These functions are regulated by hormones and require the crosstalk of the FB with other tissues. The pathways that adjust the growth rate to the nutritional conditions are the IIS and the target of rapamycin (TOR) pathways, and those involved in the systemic immunity are the Toll and Immune deficiency (Imd) pathways. In the next sections, we review the current knowledge about the role of these signalling pathways and the main factors involved in the different functions of the larval FB and in its communication with other tissues.

### Store and Release of Energy Reserves

Similar to the mammalian WAT, the *Drosophila* larval FB stores and releases energy in response to the organism energetic demands. The energy is stored mainly in the form of glycogen and of TAGs, the lipolysis products of those being transported to other tissues to support growth and survival.

#### Carbohydrates

In *Drosophila*, glycogen is the main storage form of carbohydrates and is found in the body wall muscles and in the FB in late larval stages (Baker and Thummel, 2007; Garrido et al., 2015). In addition to glycogen, trehalose is synthesized in the FB and released into the hemolymph. Upon starvation, glycogen is mobilized to maintain the circulating sugar levels (Mattila et al., 2015; Yamada et al., 2018). In mammals, the sensing of sugar at intracellular levels is mediated by the heterodimer formed by the conserved bHLH-Zip transcription factors ChREBP (Carbohydrate Response Element Binding Protein) and MondoA, together with their common partner Mlx (Max-like protein X), which are activated by sugars and promote the conversion of sugars to lipids. They control most of the sugar-responsive genes as well as carbohydrate, amino acid and lipid metabolism (Havula and Hietakangas, 2012; Mattila et al., 2015). In *Drosophila,* the single orthologs of ChREBP/Mondo and Mlx are Mondo and Bigmax, respectively, and this transcriptional network is essential for sugar tolerance also in this organism. Accordingly, the Mondo-Bigmax deficient *Drosophila* larvae presents lethality on any diet containing high levels of sucrose, glucose or fructose (Havula et al., 2013). In addition to the regulation of metabolic genes, Mondo-Bigmax regulate the expression of the TGFβ/Activin ligand Dawdle (Daw), the Gli-similar transcription factor Sugarbabe and the orthologue of mammalian Kruppel-like factors 10 and 11, Cabut (Bartok et al., 2015; Mattila et al., 2015). As detailed in next sections, the intracellular glucose sensing by Mondo-Bigmax is coupled to systemic growth through Daw. Other nutrient sensors involved in sugar tolerance are the nuclear receptor Hnf4 (Hepatocyte nuclear factor 4) and the NAD^+^-dependent deacetylase Sirtuin 1 and 2 (Sirt1, Sirt2). Hnf4 plays a critical role in carbohydrate metabolism as shown by the *Hnf4* mutant larvae, which display highly elevated circulating glucose and trehalose levels and defects in lipid homeostasis (Palanker et al., 2009; Palu and Thummel, 2016). Sirt2, is required in the FB to maintain glucose homeostasis and peripheral insulin sensitivity by deacetylating and stabilizing Hnf4 through protein interactions (Palu and Thummel, 2016). Moreover, Sirt1 negatively regulates TAG accumulation in the larval FB (Reis et al., 2010).

#### Lipids

TAG is the main lipid form in the FB, which is synthesized from dietary carbohydrates, fatty acids or proteins and is stored in intracellular LDs. Similarly to mammals, LDs of different sizes belong to distinct functional classes, which differ in their properties owing to differential association with particular sets of LD-associated proteins (Wilfling et al., 2013). Characterization of the LD proteome at different stages uncovered that LD-associated proteins are different according to the functional complexity among LDs (Cermelli et al., 2006; Beller et al., 2010; Walther and Farese, 2012; Krahmer et al., 2013). The best characterized LD proteins in the FB during the larval life are Lsd-1 and Lsd-2 (Lipid storage droplet-1 and -2), homologous to the mammalian PAT domain protein family (Perilipin, ADRP, and TIP47) (Grönke et al., 2003; Teixeira et al., 2003). Lsd-2 is required for storage of TAG, whereas Lsd-1 stimulates TAG hydrolysis (Bi et al., 2012). The subproteome analysis of LDs of *Drosophila* FB identified 248 proteins (Beller et al., 2006). Most of them were involved in cellular metabolism but proteins have been identified with diverse biological functions, including intracellular transport, cell organization and cell biogenesis. For instance, the droplet-associated protein Sturkopf has a role in endocrine physiology regulation (Werthebach et al., 2019). The *sturkopf* mutant adults show a mild decrease in TAG storage levels. However, they fail to adjust their developmental rate to dietary yeast-to-sugar ratio changes, suggesting a function in insulin and juvenile hormone signalling activities. Moreover, distinct spatially LD populations have been described in *Drosophila* FB: the peripheral LDs, in contact with the plasma membrane, and the larger cytoplasmic medial LDs. The peripheral LD homeostasis is regulated by Snazarus (Snz), which binds to LDs and promotes TAG storage (Ugrankar et al., 2019).

Interestingly, the regulation of lipid homeostasis is coupled to FB development and growth. For example, Lipin, which converts phosphatidate to diacylglycerol, is required for normal FB development and TAG storage (Ugrankar et al., 2011). Loss of *Lipin* in *Drosophila* leads to severe defects in the development of the FB with changes in cell nucleus, mitochondria, autophagosome formation and size of LDs. Similarly, the *Drosophila* BMP-5,7 orthologue, glass bottom boat (gbb), is also required for the development of the larval FB and for maintaining proper metabolism. *gbb* mutants exhibit developmental delay and altered FB morphology with reduced total lipid, glucose and trehalose levels (Ballard et al., 2010). A recent study shows that the FB expression of the atypical MAP kinase, Erk7 (Extracellularly regulated kinase 7), inhibits cell autonomous and systemic growth and lipid storage. *Erk7* expression is upregulated by fasting and, therefore, contributes to the adaptation to nutrient shortage. Erk7 regulates the subcellular localization of the chromatin binding protein No child left behind (Nclb), a growth-promoting downstream effector of mTOR, and inhibits the expression of the lipogenic transcription factor gene *sugarbabe* (Hasygar et al., 2021).

#### The Insulin/Glucagon Axis

The energy storage in the FB during the larval development is required during low nutrient conditions and for the survival during the non-feeding periods, such as before and during metamorphosis and during the early stages of adulthood. The maintenance of the metabolic homeostasis requires the communication between the nutrient-storing FB and the consuming tissues.

In mammals, the main hormones that regulate the mobilization of fat and glucose are insulin and glucagon (Freychet P. 1990). Insulin is secreted by pancreatic β cells in response to high blood sugar levels, which triggers glycogen synthesis. Under low sugar levels pancreatic α cells release glucagon and triggers the breakdown of glycogen. Glucagon is also a lipolytic hormone that regulates fatty acids, ketone bodies and TAG.

In *Drosophila*, the insulin/glucagon axis is well conserved and involves the insulin-like peptides (Ilps) and the glucagon-like peptide Akh (Schaffer et al., 1990; Semaniuk et al., 2021). The mobilization of carbohydrate and lipid energy reserves from the FB in response to starvation is regulated by Akh/AkhR, which is produced by the neurosecretory cells of the corpora cardiaca (Kim and Rulifson, 2004; Lee and Park, 2004; Isabel et al., 2005). For carbohydrate mobilization, Akh/AkhR stimulates, through glycogen phosphorylase, the conversion of stored glycogen to hemolymph trehalose, which is important during the nonfeeding periods and during adult flight. The lipid mobilization through the action of Akh/AkhR, led to the phosphorylation of Lsd-1, which activates the Triglyceride Lipase (TGL) (Arrese et al., 2006; Arrese and Soulages, 2010). However, the role of Akh/AkhR is not completely elucidated as some reports suggest that Akh/AkhR is dispensable for lipid homeostasis in third instar larvae (Lee and Park, 2004; Gáliková et al., 2015). A recent report shows that, although in nutrient abundant conditions Akh/AkhR is dispensable during larval development, in low nutrient stress conditions Akh/AkhR signalling alters larval development and the adult metabolism and behaviour (Hughson et al., 2021).

In mammals, the mobilization of fatty acids from TAG storage is coordinated by the hormone-sensitive lipase (HSL) and the Patatin Like phospholipase Domain Containing 2 (PNPLA2, also known as ATGL) (Zimmermann et al., 2004; Young and Zechner, 2013). Interestingly, ATGL-dependent lipolysis of WAT triggers a systemic insulin release, which is essential for the replenishment of BAT energy storage in mice (Heine et al., 2018). In *Drosophila*, independently of Akh/AkhR signalling, the Brummer (Bmm) lipase, homolog of mammalian ATGL, converts the accumulated TAG to fatty acids (Grönke et al., 2005).

### Nutrient Sensor and Systemic Growth

The FB acts as a sensing organ that coordinates the metabolic and physiological responses to the nutrient status of the organism. The FB relays the nutrient information through the secretion of humoral factors to the insulin-producing cells (IPCs), which secrete Ilps to control the systemic ISS.

#### Signalling Pathways in the Fat Body Regulating Body Growth

In *Drosophila* FB, the IIS and the TOR pathways regulate nutrient uptake, storage and metabolism. In addition, there is a crosstalk between the steroid hormone 20-hydroxyecdysone (ecdysone) and those pathways. Furthermore, the FB is the main sensor of internal oxygen levels that control organismal growth.

The *Drosophila* genome encodes eight Ilps (Grönke et al., 2010): Ilp, 2, 3 and 5 are produced by IPCs in the brain and are functionally comparable to insulin; Ilp6, produced by the FB, is related to mammalian Insulin Growth Factors, IGFs (Okamoto et al., 2009; Slaidina et al., 2009); Ilp7 and Ilp8 are relaxin-like peptides (Grönke et al., 2010). Similar to mammalian insulin, Ilps are able to regulate circulating levels of carbohydrates in the hemolymph. Insulin is a positive regulator of fat cell mass, acting through changes in both cell number and lipid storage (DiAngelo and Birnbaum, 2009). Ilp2 and Ilp5 regulate glycogen deposition, Ilp3 is responsible for the synthesis and release of trehalose into hemolymph and Ilp5 and Ilp7 regulate the synthesis of TAG (Kim and Rulifson, 2004; Post et al., 2018; Semaniuk et al., 2021). In addition, IIS/PI3K (Phosphatidylinositol 3-kinase) signalling coordinates nutritional status with endoreplicating tissues metabolism and growth (Britton et al., 2002). Thus, insulin regulates the critical weight, a checkpoint that occurs early in third instar larvae that determines the final body size (Mirth and Riddiford, 2007).

Mammals and *Drosophila* use the TOR pathway for cellular nutrient sensing, playing an important role in the balance of energy storage. The TOR kinase activity depends on amino acid availability and mediates protein synthesis, amino acid import, ribosome biogenesis and autophagy (Saxton and Sabatini, 2017). Consequently, *Tor* mutant larvae show reduced size and glucose and lipid storage levels, larvae showing a transparent phenotype (Colombani et al., 2003; Luong et al., 2006).

In addition, there is crosstalk between IIS and ecdysone. Ecdysone signalling in the FB antagonizes IIS and promotes autophagy (Rusten et al., 2004; Colombani et al., 2005). Furthermore, ecdysone modulates organismal growth through a FB relay that attenuates systemic insulin signalling (Colombani et al., 2005; Arquier et al., 2008; Honegger et al., 2008; Jin et al., 2012; Lee et al., 2018).

#### Humoral Fat Body Derived Signals

In *Drosophila* and other animals, the organisms require sensing the levels of oxygen to adapt their systemic growth to the environmental conditions. A central regulator for the maintenance of oxygen homeostasis is the hypoxia-inducible factor 1 (HIF-1), a heterodimeric transcription factor composed of the oxygen regulated HIF-1α and the constitutively expressed HIF-1β. In presence of oxygen, HIF-1α is hydroxylated by HIF prolyl hydroxylase (Hph), targeting it for ubiquitin-dependent proteasomal degradation. In hypoxia, HIF-1α is stabilized and induces the expression of target genes that regulate growth and metabolism (Semenza, 2014).

To link the organismal growth to the nutrient availability, the FB produces signalling molecules that promote or inhibit the insulin secretion from IPCs **(** Figure 3 and Table 3 **).** Some of these factors and neuropeptides are secreted in response to dietary fats and/or sugars such as Unpaired 2 (Upd2), Daw and CCHamide-2 (CCHa2).

Upd2, a JAK/STAT cytokine (Rajan and Perrimon, 2012), binds to its receptor Dome (Domeless) on GABAergic neurons, releases the inhibition of IPCs and promotes Ilp secretion. Recently, an essential role for adipose p53 in sensing nutrient stress and maintaining metabolic homeostasis has been reported (Ingaramo et al., 2020). Under nutrient deprivation and high-sugar diet, p53 is activated in the FB and represses the expression of Upd2. This AMP-activated protein kinase (AMPK)-dependent p53 activation leads to modulation of Ilp2 levels, systemic insulin/TOR signalling and autophagy induction (Ingaramo et al., 2020). Another response to the consumption of sugar is the release by the FB of the activin-like factor Daw, which promotes the secretion of Ilps through the TGF-β/activin receptor Baboon (Babo) (Ghosh and O’Connor, 2014). In addition, Daw released from the FB signals to the intestine where inhibits the expression of carbohydrases and lipases by enhancing Smad on X (Smox) levels (Chng et al., 2014). The sugar induced gene expression of Daw is mediated by Mondo-Bigmax, whereas Foxo (forkhead box, sub-group O) negatively regulates its expression (Bai et al., 2013; Mattila et al., 2015). A third mechanism by which carbohydrates promote Ilp expression and secretion is through CCHa2, a neuropeptide induced in the FB by proteins and sugars. When released, the CCHa2 peptide promotes the secretion of Ilp2 and Ilp5 via its receptor, CCHa2R, expressed in the IPCs (Ren et al., 2015; Sano et al., 2015).

TOR-dependent FB humoral signals couple Ilp2 and Ilp5 secretion from the IPCs with amino acid intake and some humoral factors are secreted in response to dietary amino acids such as Stunted (Sun), Eiger (Egr) and the Growth blocking peptides GBP1 and GBP2 (Colombani et al., 2003; Honegger et al., 2008; Géminard et al., 2009; Rajan and Perrimon, 2012; Ghosh and O’Connor, 2014; Sano et al., 2015; Agrawal et al., 2016; Delanoue et al., 2016; Koyama and Mirth, 2016). Interestingly, amino acid-dependent TOR signalling derived from the FB controls neural stem cell proliferation independent from IPCs-derived Ilps. In the developing central nervous system, embryonic and larval neuroblasts undergo proliferative phases, intercalated with periods of a quiescent state, that is reversible by dietary amino acids (Britton and Edgar, 1998). The TOR-mediated amino acid sensing induces a secreted FB signal that activates the expression of Ilps in glial cells. The local glial Ilps signal on adjacent neuroblasts via the IIS/PI3K/TOR pathway and control their reactivation (Chell and Brand, 2010; Sousa-Nunes et al., 2011).

Furthermore, ecdysone signalling in the FB modulates insulin dependent systemic growth through the regulation of Myc, microRNA miR-8 and ImpL2 (Ecdysone-inducible gene L2), a member of the immunoglobulin superfamily homolog to the Insulin-Like Growth Factor Binding Protein 7, IGFBP7 (Arquier et al., 2008; Honegger et al., 2008; Hyun et al., 2009; Delanoue et al., 2010; Jin et al., 2012; Lee et al., 2018).

The FB is a sensor tissue for amino acid levels and coordinates growth of peripheral tissues through a humoral mechanism (Colombani et al., 2003). Hence, the downregulation of the Slimfast (Slif) amino acid transporter within the FB is sufficient to induce a general reduction in the rate of larval growth (Colombani et al., 2003). In response to dietary amino acids, the peptide Sun is released from the FB (Delanoue et al., 2016). Sun binds to Methuselah (Mth), a secretin-incretin receptor on IPCs, and stimulates the secretion of Ilps. On the other hand, under conditions of low amino-acid concentrations, Egr, a *Drosophila* tumor necrosis factor alpha (TNF-alpha) orthologue is released from the larval FB (Agrawal et al., 2016). This cytokine signals through its receptor Grindelwald (Grnd) on the larval IPCs to activate the JNK-dependent inhibition of Ilps production. The expression of the endoplasmic reticulum (ER) degradation enhancing α-mannosidase-like protein 1 (Edem1) in the FB is also crucial for maintaining systemic insulin signalling, since its down-regulation results in the accumulation of Ilp2 in the IPCs and reduced systemic insulin signalling. The reduction in Edem1 levels is crucial for survival during starvation as lowering *edem1* expression levels facilitates the activation Eiger on IPCs and the reduction in ISS. In addition, Edem1 regulates Upd2 to manage the metabolic status (Pathak and Varghese, 2021). Moreover, Growth-blocking peptides 1 and 2 (GBP1 and GBP2) are epidermal growth factors-like cytokines secreted by the FB upon availability of dietary amino acids (Koyama and Mirth, 2016). Recently, it was shown that these adipose tissue factors regulate Ilps secretion by silencing a pair of inhibitory neurons that synapse with IPCs (Meschi et al., 2019).

During late larval life, increased levels of ecdysone affect also systemic growth. Myc expression in the *Drosophila* FB triggers a cell autonomous mechanism that controls glucose and lipid metabolism to favour the storage of nutrients (Parisi et al., 2013). During the late third instar, ecdysone signalling represses Myc function inhibiting systemic growth. This suggests a humoral factor released downstream of Myc that relays information to control IIS (Delanoue et al., 2010). The ability of FB Myc activity to affect IPC Ilp2 secretion depends on stearoyl-CoA desaturase (Desat1) activity, an enzyme necessary for production of fatty acids and lipid biosynthesis (Parisi et al., 2013). The increased levels of ecdysone suppress the body growth also through the regulation of FB microRNA miR-8 (Hyun et al., 2009; Jin et al., 2012). Multiple peptide hormones regulated by miR-8 may contribute to *Drosophila* growth (Lee et al., 2015). Among them, the IGF-like factor Ilp6 and the Imaginal morphogenesis protein Late 2 (ImpL2) are upregulated in the FB of miR-8 null mutant larvae. Ilp6 expression from larval FB represses secretion of Ilp2 from IPCs and extends lifespan (Bai et al., 2012). Before and during pupariation or in response to starvation, Ilp6 communicates the FB with other organs. For example, it promotes the growth of imaginal discs, which gives rise to adult organs, and the lipid uptake in oenocytes, cell clusters of ectodermal origin that regulate lipid metabolism (Chatterjee et al., 2014). Nutritional restriction also increases the levels of ecdysone, which triggers the production of ImpL2 in the FB (Lee et al., 2018). In response to nutrient limitation, the FB nutrient sensor function, which restricts the growth of peripheral tissues, is complemented by the release of nutrients through autophagic degradation of the FB cytoplasm. This provides other tissues with a source of nutrients necessary for survival. Thus, under conditions of low TOR signalling, autophagy promotes normal cell function and survival (Scott et al., 2004).

To adapt the systemic growth to the environmental conditions, the FB integrates the oxygen and amino acids levels through the Hph/HIF-1α and Hph/TOR pathways. In hypoxia, the FB release HIF-1α-dependent humoral factors that inhibit Ilps expression and secretion from the IPCs, thereby restricting the systemic growth. Moreover, independently of HIF-1α, Hph is required for nutrient-dependent TOR activation (Texada et al., 2019). To allow adults viability in hypoxia, the larval FB inhibits TORC1 signalling and reorganizes the lipid storage (Lee et al., 2019). A recent study showed that FOXO is a hypoxia inducible factor that mediates tolerance to low oxygen by inducing immune-like responses in the FB (Barretto et al., 2020).

### Systemic Immunity

The *Drosophila* FB coordinates not only the nutrient storage and the animal growth but also the humoral immune response. In *Drosophila*, the infection by microbes induces the secretion of antimicrobial peptides (AMP) by the FB, which are controlled by the Toll and Imd pathways (De Gregorio et al., 2002). The Toll-NF-kB signalling, which triggers the nuclear translocation of Dif (Dorsal-related immunity factor) and Dorsal, is induced by fungi and Gram-positive bacteria, whereas infection by Gram-negative bacteria leads to the processing and transport or Relish via the Imd pathway. To support the immune activation, the FB increases its volume, expands the ER and alters its metabolism, shifting from lipid metabolism to membrane phospholipid synthesis (Martínez et al., 2020). These changes, induced by Toll signalling to sustain AMP synthesis and secretion, may become detrimental if maintained over long periods due to insufficient nutrient storage. Thus, the expression of a constitutively active Toll receptor in the larval FB inhibits the whole organismal growth, disrupts the insulin signalling in the FB and reduces the TAG storage (DiAngelo and Birnbaum, 2009; Roth et al., 2018; Suzawa et al., 2019). Similarly, persistent activation of the Imd pathway in the larval FB diminished IIS/TOR activity, which resulted in decreased TAG levels and impaired whole animal growth (Davoodi et al., 2019). Moreover, increasing insulin signalling in the FB leads to decreased immune gene expression and, vice versa, decreasing insulin signalling leads to increased immune gene expression and increased resistance to infection (Musselman and Kühnlein, 2018). This supports a model in which insulin signalling and the immune response negatively regulate each other to maintain the energy balance.

## Origin and Development/Differentiation of Adult Fat Body

The origin of the adult FB, in invertebrate, as in mammals, remains elusive and unexplored due to the difficulty in its manipulation (The mesoderm and its derivatives and BM, 1993; Hoshizaki et al., 1995; Berry et al., 2013). Although both larval and adult FBs play a role as energy storage organs and nutrient availability sensing, they show different features. For example, contrary to the larval FB, adult FB is able to expand by increasing the number of adipocytes. Moreover, they might not share a common origin: while larval FB derives from the nine embryonic bilateral primordia, the origin of the adult FB has not been identified (Hoshizaki et al., 1994; Hoshizaki et al., 1995; Aguila et al., 2007).

During metamorphosis, unlike most larval tissues that undergo histolysis, some of the larval FB cells persist and are found in the newly eclosed adult, free floating as single cells or small clusters. These larval fat cells are refractive to the autophagic cell death that removes most of the larval cells during metamorphosis. It has been shown that these larval adipocytes, now dissociated, are a source of nutrients during the non-feeding stage of adulthood, approximately the first 3 days after eclosion (Aguila et al., 2007). Three to five days after eclosion these cells are replaced by the adult fat adipocytes (Johnson and Butterworth, 1985), which accumulate lipid reserves through feeding and *de novo* lipid synthesis during those days. The myokine Pvf1 (PDGF- and VEGF-related factor 1) represses lipid synthesis at the end of the adult FB lipid build-up phase by activating TOR pathway specifically in the oenocytes (Ghosh et al., 2020). Adult adipocytes must develop from some pupal progenitors, the specific cells that give rise to the adult fat cells have not been identified (The mesoderm and its derivatives and BM, 1993).

The development of adult FB might require the expression of genes driven by a number of enhancers that are identified through enhancer traps (Table 4). Most of them, except for 29D and *RD61*, drive the expression in larval FB as well as in adult one *3-76a*, *X8-157a* and *l(3)2E2* driving the expression in fat cells of all stages. As *l(3)2E2* is an enhancer of the *srp* gene, the activity of Srp might be also involved in fat cell decision or/and differentiation programmes at adult stage **(** Tables 3, 4 **)** (Hoshizaki et al., 1995).

Although the cells that give rise to the adult fat cells have not been identified, two fundamentally different mechanisms have been suggested to explain how the adult FB arises: 1) cell remodelling, a process in which larval FB tissue is dissociated into isolated cells that later associate to form the adult FB (Larsen, 1976), or 2) the complete destruction of larval FB and simultaneous synthesis of adult FB from undifferentiated ASC (Haunerland and Shirk, 1995).

### Potential Adipose Stem Cell Population

It has been shown that adult FB derives from the mesoderm (Lawrence and Johnston, 1986). However, the ASC population that maintain the adult FB has not been identified. Hoshizaki *et al.* suggested that a subset of adepithelial cells, precursors of adult thoracic muscles, might be as well the precursors of adult adipocytes (Hoshizaki et al., 1995). Adepithelial cells are in fact a plausible source of ASCs, since two of the fat cells-specific enhancer traps mentioned above, *3-76a* and *X8-157a*, are also active in the adepithelial cells. This suggests a possible lineage connexion for fat cells from embryo to adult, including the adepithelial cells during larval stage. Furthermore, adepithelial cells are the precursors of adult muscles (Holz et al., 1997) and a subset of these cells expressing Breathless are the precursors of the adult tracheal air sacs (Sato and Kornberg, 2002). This might suggest that adepithelial cells could potentially be the pluripotent stem cell population in the adult stage.

## Role of Adult Fat Body

In spite of the fact that most of the functional studies in *Drosophila* are conducted at larval stages, there are enough evidences to ensure that the adult FB carries out liver, adipose, and immune functions (Hotamisligil, 2017; Arrese and Soulages, 2010). Oenocytes, specialized hepatocyte-like cells, are closely associated to adipocytes, specifically at the subcuticular FB (Figure 2) (Gutierrez et al., 2007). In fact, very recently, the role of oenocytes regulating lipid synthesis and content in the adipose tissue has been described, a role-played by hepatocytes in mammals. Furthermore, loss of function of TOR pathway in adult oenocytes leads to obesity (Ghosh et al., 2020).

Although further studies would be necessary to prove the equivalence of these organs, there is a subcuticular FB that is extended through the whole *Drosophila* adult body (Figure 2), and also a FB wrapping some organs such as the heart, intestine, spermatheca and brain, and these could be the equivalent to mammalian subcutaneous and visceral WAT, respectively.

There are not many evidences indicating the existence of a BAT or beige adipocytes tissue in *Drosophila*. However, a set of genes coding for Uncouple proteins, including UCP1 that is a marker for BAT in mammalian systems, are conserved in *Drosophila* (Harms and Seale, 2013). Similarly to UCP1, *Drosophila* Ucp4C has been involved into the dissipation of energy in the mitochondria (Cannon and Nedergaard, 2004; Da-Ré et al., 2014).

### Metabolism

**FIGURE 4:**
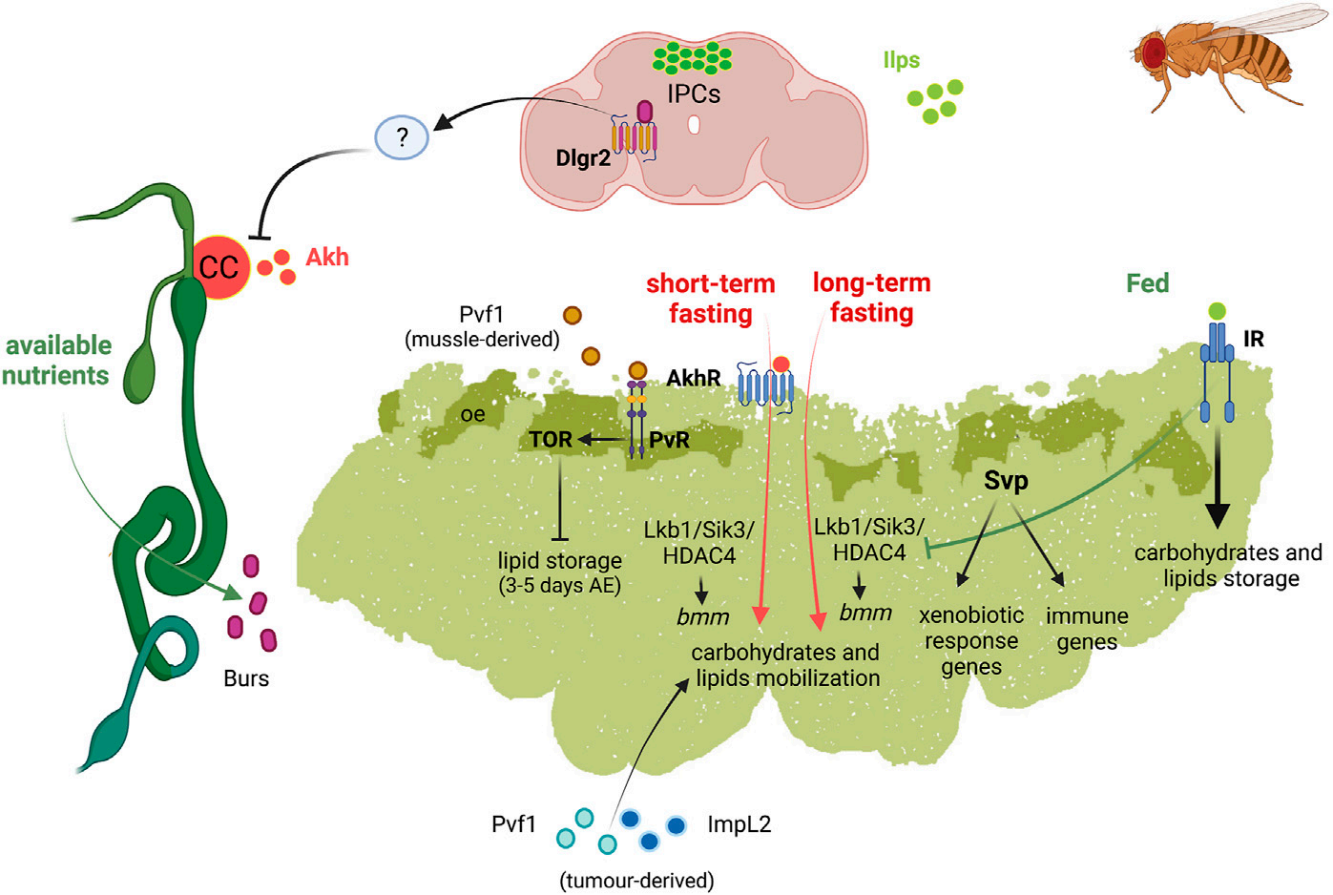
Schematic representation of the diverse functions of *Drosophila* fat body in adult fly. Left, adult digestive system represented in green; top, adult brain represented in soft purpure. Abbreviations: Akh Adipokinetic hormone; AkhR, Akh receptor; bmm, brummer lipase gene; Burs, Bursicon receptor; CC, corpora cardiaca; Dlgr2, leucine rich repeat containing G protein-coupled receptor 2; HDAC4, Histone desacetylase 4; ImpL2, Imaginal morphogenesis protein-late 2; Ilps, insulin-like peptides; IPC, insulin producing cells; IR, insulin receptor; Lkb1, Liver kinase B1; oe, oenocytes; Pvf1, PDGF- and VEGF-related factor 1; PvR, Pvf1 receptor; Sik3, Salt-inducible kinase 3; Svp, Seven up; TOR, target of rapamycin.

Adult FB has an important role in physiology, longevity as well as disease, e.g., cancer (Figure 4)**.**

#### Metabolic Organ

Similarly to the larval one, the adult FB is the central metabolic organ involved in the accumulation of fat and glycogen from caloric overload and in the mobilization of the stored fat during starvation or egg production (Lee and Park, 2004; Parra-Peralbo and Culi, 2011; Musselman et al., 2013; Mattila and Hietakangas, 2017; Zhao and Karpac, 2017; Weaver and Drummond-Barbosa, 2019). Not surprisingly, in females the FB has higher proportion of lipids than that in males (Johnson and Butterworth, 1985). In contrast to larval FB there are evidences suggesting the ability of adult FB to grow in order to accumulate lipids, in obese flies (DiAngelo and Birnbaum, 2009).

In the adult FB, Akh/AkhR signalling activates cAMP-responsive element binding (CREB) transcription factor (Song et al., 2017). CREB downregulation was shown to promote overeating and obesity in adult flies (Iijima et al., 2009). In addition, Akh/AkhR signalling modulates TAG content in adult FB through the store-operated calcium entry (SOCE) (Baumbach et al., 2014). Under short-term fasting conditions, Akh/AkhR signalling promotes lipase *bmm* gene expression by reducing Lkb1-Sik3 (Salt-inducible kinase 3)-HDAC4 (Histone deacetylase 4) signalling axis, probably through Foxo (Wang et al., 2011; Choi et al., 2015). Under long-term fasting conditions, however, the reduction of the Lkb1-Sik3 pathway to induce the lipolytic response is independent of Akh/AkhR. Conversely, insulin pathway induces Sik3 activity under feeding conditions, independently of Lkb1 (Choi et al., 2015) (Figure 4).

Recently, Relish known to be part of Imd pathway, as mentioned above, has been identified as a repressor of *bmm* gene expression through FOXO, by fasting-dependent histone deacetylation, during metabolic adaptation to fasting (Molaei et al., 2019).

#### Crosstalk in Inter-Organ Communication: Links to Fat Body

Recently, an intestinal/neuronal/FB inter-organ communication has been described in adults to preserve energy homeostasis. In response to nutrients, enteroendocrine cells secrete systemically the hormone Bursicon α (Bursα), which binds to its neural receptor DLgr2. Bursα/DLgr2 signalling regulates energy metabolism through a neuronal relay that repress AKH production and, therefore, the subsequent modulation of AKHR signalling within the FB. The reduction of systemic Bursα/DLgr2 signalling leads to exacerbated glucose oxidation, strong lipodystrophy and depletion of energy stores with the consequent reduced organismal resistance to nutrient deprivation conditions (Scopelliti et al., 2019). Therefore, Bursicon inhibits the mobilization of glycogen storage under nutrient availability (Figure 4).

#### Aging and Longevity

The overexpression in the adult FB of the gene *foxo*, encoding for the key target of the IIS pathway, leads to increased life span (Giannakou et al., 2004). Similarly, overexpression of the gene *Sirt2* in adult FB increases longevity in both sexes. It also modulates the composition of the LD proteome, a plausible mechanism underlying extended longevity by *Sirt2*, as LDs regulate aging processes (Goldberg et al., 2009; Hoffmann et al., 2013). All these evidences point to a role of the adult AT in controlling longevity.

#### Cancer-Associated Cachexia

Tumors and their microenvironment can produce different circulating factors that cause cachexia, the wasting syndrome observed in advance cancer patients which is characterized by a general metabolic dysfunction that includes systemic inflammation, increased catabolism and lipolysis or proteolysis in muscles and AT (Ebadi and Mazurak, 2014). In *Drosophila,* two main models of cachexia have been described which show similarities with human patients (Figueroa-Clarevega and Bilder, 2015; Kwon et al., 2015; Song et al., 2019). One of the models is induced by activation of Yorkie (Yki), the Yap1 oncogen ortholog, in intestine stem cells that secrete a PDGF-and VEGF-related factor 1 (PvF1) ligand. Pvf1 leads to the pathological activation of ERK/MAPK signalling in peripheral tissues and induce wasting of muscles and AT (Song et al., 2019). The other model consists of the transplantation, in adult flies, of clones of eye disc cells mutant for the polarity gene *scribble* and ectopically expressing an activated form of Ras ^(V12)^ (*Ras* ^*V12*^ *, scrib* ^*−/−*^). Interestingly, both tumor models secrete high levels of ImpL2 (Figueroa-Clarevega and Bilder, 2015; Kwon et al., 2015). Increased levels of circulating ImpL2 reduce systemic insulin signalling, which leads to reduction of nutrients uptake by muscle and adipose tissue, driving organ wasting. The *Ras* ^*V12*^ *, scrib* ^*−/−*^ tumors also induce a systemic autophagy stress response in muscles and AT that mediates organ wasting (Katheder et al., 2017; Khezri et al., 20215). Recently, another wasting model in *Drosophila*, relates the FB remodelling and muscle detachment to the tumor-secreted matrix metalloproteinase 1 (Mmp1). Mmp1 can modulate TGFβ signalling in the FB and disrupts the basement membrane/extracellular matrix in FB and muscle (Lodge et al., 2021). All theses studies show that the conservation of the signalling pathways and the existing genetic tools, make of *Drosophila* an important model to study the process of organ wasting and to identify new molecular mechanisms involved in this process.

### Immunity and Xenobiotic Response

The FB acts as a detoxifying tissue based on the expression of members of the Cytochrome P450 (Cyp450) superfamily of monooxygenases. These are enzymes involved in metabolizing foreign substances and drugs implicated in resistance to insecticides (Feyereisen, 1999; Chung et al., 20097; Terhzaz et al., 2015).

Recently, Weaver and Drummond-Barbosa showed that the nuclear receptor Svp regulates a number of factors involved in immunity and xenobiotic detoxification responses in adult female FB (Weaver and Drummond-Barbosa, 2020). Specifically, Svp would acts as the first line of defence against infections, regulating genes involved in the capture and elimination of foreign pathogens. Svp also regulates the expression of genes encoding members of the CYP450 family involved in the initiation of phase I of the xenobiotic detoxification response. Reduction of *svp* expression results in the upregulation of genes encoding Metallothionein A and B (*MtnA* and *MtnB*) (Weaver and Drummond-Barbosa, 2020). MtnA and B are enzymes involved in heavy metal detoxification and protection against free radicals and have been involved in the response to xenobiotic and immune stress (Bonneton et al., 1996). It has been suggested that a reduced activity of Svp could lead to a toxic scenario, which would need MtnA and B activity to eliminate this toxicity (Weaver and Drummond-Barbosa, 2020) (Figure 4).

## Concluding Remarks and Future Perspectives

The AT is a central organ, which regulates metabolism and immune responses, as inflammation, so that it has a major impact on human physiology. AT dysfunction associates to metabolic diseases such as: obesity, diabetes, lipodystrophies and cancer-associated cachexia.

Despite of the advance in the knowledge in the last years, still there are many open questions that need to address about the functions and development of AT.

However, the knowledge at this moment can only be obtained through the studies of animal models. *Drosophila* can be a good model for the study of AT based on the possibility of the genetics analysis that can be performed *in vivo*, the lower complexity of the tissue and the functional conservation of this tissue along the evolution.

Further studies focused on tracing the cell lineages expressing the transcription factors Svp, Srp and Kr*,* involved in the determination and differentiation and maintenance of fat cells during embryonic and larval stages, would shed light on how those processes develop and what are the actors involved. Similarly, it would be very interesting to trace the cells showing expression driven by the enhancer traps *l(3)2E2*, *3-76a* and *X8-157*, which show expression in embryonic, larval and adult fat cells.

Adepithelial cells could potentially represent the ASC population of adult FB in *Drosophila*. Future characterization of the gene expression profile of this population will help to understand the origin and cellular differentiation of adult adipocytes. Also, it will reveal the mechanisms leading to the different adipocyte fates as well as putative fate-switching factors.

Most of the studies that shed light on the functions carried out by FB were conducted at larval stages. Therefore, further studies are needed to characterize and identify potentially new functions of the adult AT related to regulation of energy homeostasis and immunity that may be conserved in mammals. The identification of adult FB-secreted derived signals would drive to a comprehensive understanding of the roles that this organ is playing in inter-organ communication and in AT wasting, which would help to understand human cancer-associated cachexia and other diseases like obesity and DMT2.

## References

[B1] Agrawal N. Delanoue R. Mauri A. Basco D. Pasco M. Thorens B. (2016). The Drosophila TNF Eiger Is an Adipokine that Acts on Insulin-Producing Cells to Mediate Nutrient Response. Cell Metab 23 (4), 675–684. 10.1016/j.cmet.2016.03.003 27076079

[B2] Aguila J. R. Suszko J. Gibbs A. G. Hoshizaki D. K. (2007). The Role of Larval Fat Cells in Adult *Drosophila melanogaster* . J. Exp. Biol. 210 (6), 956–963. 10.1242/jeb.001586 17337708

[B3] Alcalá M. Calderon-Dominguez M. Serra D. Herrero L. Viana M. (2019). Mechanisms of Impaired Brown Adipose Tissue Recruitment in Obesity. Front. Physiol. Vol. 10. 10.3389/fphys.2019.00094 PMC6381290 30814954

[B4] Almind K. Manieri M. Sivitz W. I. Cinti S. Kahn C. R. (2007). Ectopic Brown Adipose Tissue in Muscle Provides a Mechanism for Differences in Risk of Metabolic Syndrome in Mice. Proc. Natl. Acad. Sci. 104 (7), 2366–2371. 10.1073/pnas.0610416104 17283342 PMC1892979

[B5] Arquier N. Géminard C. Bourouis M. Jarretou G. Honegger B. Paix A. (2008). Drosophila ALS Regulates Growth and Metabolism through Functional Interaction with Insulin-like Peptides. Cel Metab 7 (4), 333–338. 10.1016/j.cmet.2008.02.003 18396139

[B6] Arrese E. L. Patel R. T. Soulages J. L. (2006). The Main Triglyceride-Lipase from the Insect Fat Body Is an Active Phospholipase A1: Identification and Characterization. J. Lipid Res. 47 (12), 2656–2667. 10.1194/jlr.m600161-jlr200 17005997

[B7] Arrese E. L. Soulages J. L. (2010). Insect Fat Body: Energy, Metabolism, and Regulation. Annu. Rev. Entomol. 55, 207–225. 10.1146/annurev-ento-112408-085356 19725772 PMC3075550

[B8] Arthur S. T. Noone J. M. Van Doren B. A. Roy D. Blanchette C. M. (2014). One-year Prevalence, Comorbidities and Cost of Cachexia-Related Inpatient Admissions in the USA. Drugs Context 3, 212265. 10.7573/dic.212265 25126097 PMC4130358

[B9] Azeez O. I. Meintjes R. Chamunorwa J. P. (2014). Fat Body, Fat Pad and Adipose Tissues in Invertebrates and Vertebrates: The Nexus. Lipids Health Dis. Vol. 13. 10.1186/1476-511x-13-71 PMC4005007 24758278

[B10] Babaei R. Schuster M. Meln I. Lerch S. Ghandour R. A. Pisani D. F. (2018). Jak-Tgfβ Cross-Talk Links Transient Adipose Tissue Inflammation to Beige Adipogenesis. Sci. Signal. 11 (527), 11. 10.1126/scisignal.aai7838 29692363

[B11] Bachman E. S. Dhillon H. Zhang C. Y. Cinti S. Bianco A. C. Kobilka B. K. (2002). betaAR Signaling Required for Diet-Induced Thermogenesis and Obesity Resistance. Science 297 (5582), 843–845. 10.1126/science.1073160 12161655

[B12] Bai H. Kang P. Hernandez A. M. Tatar M. (2013). Activin Signaling Targeted by Insulin/dFOXO Regulates Aging and Muscle Proteostasis in Drosophila. Plos Genet. 9 (11), e1003941. 10.1371/journal.pgen.1003941 24244197 PMC3820802

[B13] Bai H. Kang P. Tatar M. (2012). Drosophila Insulin-like Peptide-6 (Dilp6) Expression from Fat Body Extends Lifespan and Represses Secretion of Drosophila Insulin-like Peptide-2 from the Brain. Aging Cell 11 (6), 978–985. 10.1111/acel.12000 22935001 PMC3500397

[B14] Baker K. D. Thummel C. S. (2007). Diabetic Larvae and Obese Flies-Emerging Studies of Metabolism in Drosophila. Cel Metab. Vol. 6, 257–266. 10.1016/j.cmet.2007.09.002 PMC2231808 17908555

[B15] Ballard S. L. Jarolimova J. Wharton K. A. (2010). Gbb/BMP Signaling Is Required to Maintain Energy Homeostasis in Drosophila. Dev. Biol. 337 (2), 375–385. 10.1016/j.ydbio.2009.11.011 19914231 PMC2838617

[B16] Barretto E. C. Polan D. M. Beevor-Potts A. N. Lee B. Grewal S. S. (2020). Tolerance to Hypoxia Is Promoted by FOXO Regulation of the Innate Immunity Transcription Factor NF-kB/relish in Drosophila. Genetics 215 (4), 1013–1025. 10.1534/genetics.120.303219 32513813 PMC7404245

[B17] Bartok O. Teesalu M. Ashwall‐Fluss R. Pandey V. Hanan M. Rovenko B. M. (2015). The Transcription Factor Cabut Coordinates Energy Metabolism and the Circadian Clock in Response to Sugar Sensing. EMBO J. 34 (11), 1538–1553. 10.15252/embj.201591385 25916830 PMC4474529

[B18] Baumbach J. Hummel P. Bickmeyer I. Kowalczyk K. M. Frank M. Knorr K. (2014). A drosophila *In Vivo* Screen Identifies Store-Operated Calcium Entry as a Key Regulator of Adiposity. Cel Metab. 19 (2), 331–343. 10.1016/j.cmet.2013.12.004 24506874

[B19] Beller M. Riedel D. Jänsch L. Dieterich G. Wehland J. Jäckle H. (2006). Characterization of the Drosophila Lipid Droplet Subproteome. Mol. Cel Proteomics 5 (6), 1082–1094. 10.1074/mcp.m600011-mcp200 16543254

[B20] Beller M. Bulankina A. V. Hsiao H.-H. Urlaub H. Jäckle H. Kühnlein R. P. (2010). PERILIPIN-dependent Control of Lipid Droplet Structure and Fat Storage in Drosophila. Cel Metab. 12 (5), 521–532. 10.1016/j.cmet.2010.10.001 21035762

[B21] Benador I. Y. Veliova M. Mahdaviani K. Petcherski A. Wikstrom J. D. Assali E. A. (2018). Mitochondria Bound to Lipid Droplets Have Unique Bioenergetics, Composition, and Dynamics that Support Lipid Droplet Expansion. Cel Metab. 27 (4), 869–885. e6. 10.1016/j.cmet.2018.03.003 PMC5969538 29617645

[B22] Berry D. C. Stenesen D. Zeve D. Graff J. M. (2013). The Developmental Origins of Adipose Tissue, 140. Cambridge): DevelopmentDevelopment, 3939–3949. 10.1242/dev.080549 PMC3775412 24046315

[B23] Bharucha K. N. Tarr P. Zipursky S. L. (2008). A Glucagon-like Endocrine Pathway inDrosophilamodulates Both Lipid and Carbohydrate Homeostasis. J. Exp. Biol. 211 (19), 3103–3110. 10.1242/jeb.016451 18805809 PMC2714167

[B24] Bi J. Xiang Y. Chen H. Liu Z. Grönke S. Kühnlein R. P. (2012). Opposite and Redundant Roles of the Two Drosophila: Perilipins in Lipid Mobilization. J. Cel Sci 125 (15), 3568–3577. 10.1242/jcs.101329 22505614

[B25] Billon N. Iannarelli P. Monteiro M. C. Glavieux-Pardanaud C. Richardson W. D. Kessaris N. (2007). The Generation of Adipocytes by the Neural Crest. Development 134 (12), 2283–2292. 10.1242/dev.002642 17507398 PMC6334830

[B26] Bonneton F. Théodore L. Silar P. Maroni G. Wegnez M. (1996). Response of Drosophila Metallothionein Promoters to Metallic, Heat Shock and Oxidative Stresses. FEBS Lett. 380 (1–2), 33–38. 10.1016/0014-5793(95)01544-2 8603742

[B27] Britton J. S. Edgar B. A. (1998). Environmental Control of the Cell Cycle in Drosophila: Nutrition Activates Mitotic and Endoreplicative Cells by Distinct Mechanisms. Development 125 (11), 2149–2158. 10.1242/dev.125.11.2149 9570778

[B28] Britton J. S. Lockwood W. K. Li L. Cohen S. M. Edgar B. A. (2002). Drosophila’s insulin/PI3-Kinase Pathway Coordinates Cellular Metabolism with Nutritional Conditions. Dev. Cel 2 (2), 239–249. 10.1016/s1534-5807(02)00117-x 11832249

[B29] Buch S. Melcher C. Bauer M. Katzenberger J. Pankratz M. J. (2008). Opposing Effects of Dietary Protein and Sugar Regulate a Transcriptional Target of Drosophila Insulin-like Peptide Signaling. Cel Metab. 7 (4), 321–332. 10.1016/j.cmet.2008.02.012 18396138

[B30] Butterworth F. M. Emerson L. Rasch E. M. (1988). Maturation and Degeneration of the Fat Body in the Drosophila Larva and Pupa as Revealed by Morphometric Analysis. Tissue Cell 20 (2), 255–268. 10.1016/0040-8166(88)90047-x 3136556

[B31] Campos-Ortega J. A. Hartenstein V. (1985). The Embryonic Development of *Drosophila melanogaster* . Springer Berlin Heidelberg.

[B32] Cannon B. Nedergaard J. (2004). Brown Adipose Tissue: Function and Physiological Significance. Physiol. Rev. Vol. 84, 277–359. 10.1152/physrev.00015.2003 14715917

[B33] Cannon B. Jong J. M. A. Fischer A. W. Nedergaard J. Petrovic N. (2020). Human Brown Adipose Tissue: Classical Brown rather Than Brite/beige? Exp. Physiol. 105 (8), 1191–1200. 10.1113/ep087875 32378255

[B34] Cermelli S. Guo Y. Gross S. P. Welte M. A. (2006). The Lipid-Droplet Proteome Reveals that Droplets Are a Protein-Storage Depot. Curr. Biol. 16 (18), 1783–1795. 10.1016/j.cub.2006.07.062 16979555

[B35] Charroux B. Royet J. (2010). Drosophila Immune Response: From Systemic Antimicrobial Peptide Production in Fat Body Cells to Local Defense in the Intestinal Tract. Fly 4 (1), 40–47. 10.4161/fly.4.1.10810 20383054

[B36] Chatterjee D. Katewa S. D. Qi Y. Jackson S. A. Kapahi P. Jasper H. (2014). Control of Metabolic Adaptation to Fasting by dILP6-Induced Insulin Signaling in Drosophila Oenocytes. Proc. Natl. Acad. Sci. U S A. 111 (50), 17959–17964. 10.1073/pnas.1409241111 25472843 PMC4273364

[B37] Chell J. M. Brand A. H. (2010). Nutrition-responsive Glia Control Exit of Neural Stem Cells from Quiescence. Cell 143 (7), 1161–1173. 10.1016/j.cell.2010.12.007 21183078 PMC3087489

[B38] Chng W. bin. A. Sleiman M. S. B. Schüpfer F. Lemaitre B. (2014). Transforming Growth Factor β/activin Signaling Functions as a Sugar-Sensing Feedback Loop to Regulate Digestive Enzyme Expression. Cell Rep 9 (1), 336–348. 10.1016/j.celrep.2014.08.064 25284780

[B39] Choi S. Lim D. S. Chung J. (2015). Feeding and Fasting Signals Converge on the LKB1-SIK3 Pathway to Regulate Lipid Metabolism in Drosophila. Plos Genet. 11 (5). 10.1371/journal.pgen.1005263 PMC4440640 25996931

[B40] Chung H. Sztal T. Pasricha S. Sridhar M. Batterham P. Daborn P. J. (2009). Characterization of *Drosophila melanogaster* Cytochrome P450 Genes. Proc. Natl. Acad. Sci. U S A. 106 (14), 5731–5736. 10.1073/pnas.0812141106 19289821 PMC2667016

[B41] Chung K.-J. Chatzigeorgiou A. Economopoulou M. Garcia-Martin R. Alexaki V. I. Mitroulis I. (2017). A Self-Sustained Loop of Inflammation-Driven Inhibition of Beige Adipogenesis in Obesity. Nat. Immunol. 18 (6), 654–664. 10.1038/ni.3728 28414311 PMC5436941

[B42] Cinti S. (2018). Adipose Organ Development and Remodeling. Compr. Physiol. Vol. 8, 1357–1431. 10.1002/cphy.c170042 30215863

[B43] Cinti S. (2012). The Adipose Organ at a Glance. Dis. Model. Mech. 5, 588–594. 10.1242/dmm.009662 22915020 PMC3424455

[B44] Cleal L. Aldea T. Chau Y.-Y. (2017). Fifty Shades of white: Understanding Heterogeneity in white Adipose Stem Cells. Adipocyte 6, 205–216. 10.1080/21623945.2017.1372871 28949833 PMC5638386

[B45] Cohen P. Spiegelman B. M. (2016). Cell Biology of Fat Storage. MBoC 27, 2523–2527. 10.1091/mbc.e15-10-0749 27528697 PMC4985254

[B46] Collins S. Daniel K. W. Petro A. E. Surwit R. S. (1997). Strain-Specific Response Toβ3-Adrenergic Receptor Agonist Treatment of Diet-Induced Obesity in Mice1. Endocrinology 138 (1), 405–413. 10.1210/endo.138.1.4829 8977430

[B47] Colombani J. Bianchini L. Layalle S. Pondeville E. Dauphin-Villemant C. Antoniewski C. (2005). Antagonistic Actions of Ecdysone and Insulins Determine Final Size in Drosophila. Science 310 (5748), 667–670. 10.1126/science.1119432 16179433

[B48] Colombani J. Raisin S. Pantalacci S. Radimerski T. Montagne J. Léopold P. (2003). A Nutrient Sensor Mechanism Controls Drosophila Growth. Cell 114 (6), 739–749. 10.1016/s0092-8674(03)00713-x 14505573

[B49] Cypess A. M. Kahn C. R. (2010). Brown Fat as a Therapy for Obesity and Diabetes. Curr. Opin. Endocrinol. Diabetes Obes. 17, 143–149. 10.1097/med.0b013e328337a81f 20160646 PMC3593105

[B50] Cypess A. M. Lehman S. Williams G. Tal I. Rodman D. Goldfine A. B. (2009). Identification and Importance of Brown Adipose Tissue in Adult Humans. N. Engl. J. Med. 360 (15), 1509–1517. 10.1056/nejmoa0810780 19357406 PMC2859951

[B51] Cypess A. M. White A. P. Vernochet C. Schulz T. J. Xue R. Sass C. A. (2013). Anatomical Localization, Gene Expression Profiling and Functional Characterization of Adult Human Neck Brown Fat. Nat. Med. 19 (5), 635–639. 10.1038/nm.3112 23603815 PMC3650129

[B52] Da-Ré C. De Pittà C. Zordan M. A. Teza G. Nestola F. Zeviani M. (2014). UCP4C Mediates Uncoupled Respiration in Larvae of *Drosophila melanogaster* . EMBO Rep. 15 (5), 586–591. 24639557 10.1002/embr.201337972 PMC4210097

[B53] Daum G. Wagner A. Czabany T. Athenstaedt K. (2007). Dynamics of Neutral Lipid Storage and Mobilization in Yeast. Biochimie 89, 243–248. 10.1016/j.biochi.2006.06.018 16919863

[B54] Davoodi S. Galenza A. Panteluk A. Deshpande R. Ferguson M. Grewal S. (2019). The Immune Deficiency Pathway Regulates Metabolic Homeostasis in Drosophila. J. Immunol. 202 (9), 2747–2759. 10.4049/jimmunol.1801632 30902902

[B55] De Gregorio E. Spellman P. T. Tzou P. Rubin G. M. Lemaitre B. (2002). The Toll and Imd Pathways Are the Major Regulators of the Immune Response in Drosophila. EMBO J. 21 (11), 2568–2579. 10.1093/emboj/21.11.2568 12032070 PMC126042

[B56] Dean R. L. Locke M. Collins J. V. (1985). “Structure of the Fat Body,” in Comprehensive Insect Physiology, Biochemistry, and Pharmacology. Editors Kerkut G. A. Gilbert L. I. (Oxford: Pergamon Press), Vol. 3, 155–210. 10.1016/b978-0-08-030804-3.50011-x

[B57] Delanoue R. Meschi E. Agrawal N. Mauri A. Tsatskis Y. McNeill H. (2016). Drosophila Insulin Release Is Triggered by Adipose Stunted Ligand to Brain Methuselah Receptor. Science 353 (6307), 1553–1556. 10.1126/science.aaf8430 27708106

[B58] Delanoue R. Slaidina M. Léopold P. (2010). The Steroid Hormone Ecdysone Controls Systemic Growth by Repressing dMyc Function in drosophila Fat Cells. Dev. Cel 18 (6), 1012–1021. 10.1016/j.devcel.2010.05.007 20627082

[B59] DiAngelo J. R. Birnbaum M. J. (2009). Regulation of Fat Cell Mass by Insulin in *Drosophila melanogaster* . Mol. Cel Biol 29 (24), 6341–6352. 10.1128/mcb.00675-09 PMC2786867 19822665

[B60] Ding H. Zheng S. Garcia-Ruiz D. Hou D. Wei Z. Liao Z. (2016). Fasting Induces a Subcutaneous-To-Visceral Fat Switch Mediated by microRNA-149-3p and Suppression of PRDM16. Nat. Commun. 7, 11533. 10.1038/ncomms11533 27240637 PMC4895052

[B61] Doane W. W. (1960). Developmental Physiology of the Mutantfemale Sterile(2)adipose ofDrosophila Melanogaster. I. Adult Morphology, Longevity, Egg Production, and Egg Lethality. J. Exp. Zool. 145 (1), 1–21. 10.1002/jez.1401450102 13723227

[B62] Doria A. Patti M.-E. Kahn C. R. (2008). The Emerging Genetic Architecture of Type 2 Diabetes. Cel Metab. 8, 186–200. 10.1016/j.cmet.2008.08.006 PMC4267677 18762020

[B63] Ebadi M. Mazurak V. C. (2014). Evidence and Mechanisms of Fat Depletion in Cancer. Nutrients Vol. 6, 5280–5297. 10.3390/nu6115280 PMC4245589 25415607

[B64] Enerbäck S. (2009). The Origins of Brown Adipose Tissue. N. Engl. J. Med. 360 (19), 2021–2023. 19420373 10.1056/NEJMcibr0809610

[B65] Enomoto M. Siow C. Igaki T. (2018). Drosophila as a Cancer Model. Adv. Exp. Med. Biol., 173–194. 10.1007/978-981-13-0529-0_10 29951820

[B66] Evans C. J. Olson J. M. Ngo K. T. Kim E. Lee N. E. Kuoy E. (2009). G-TRACE: Rapid Gal4-Based Cell Lineage Analysis in Drosophila. Nat. Methods 6 (8), 603–605. 10.1038/nmeth.1356 19633663 PMC2754220

[B67] Fearon K. Strasser F. Anker S. D. Bosaeus I. Bruera E. Fainsinger R. L. (2011). Definition and Classification of Cancer Cachexia: An International Consensus. Lancet Oncol. 12, 489–495. 10.1016/s1470-2045(10)70218-7 21296615

[B68] Feldmann H. M. Golozoubova V. Cannon B. Nedergaard J. (2009). UCP1 Ablation Induces Obesity and Abolishes Diet-Induced Thermogenesis in Mice Exempt from Thermal Stress by Living at Thermoneutrality. Cel Metab. 9 (2), 203–209. 10.1016/j.cmet.2008.12.014 19187776

[B69] Feyereisen R. (1999). Insect P450 Enzymes. Annu. Rev. Entomol. Vol. 44, 507–533. 10.1146/annurev.ento.44.1.507 9990722

[B70] Figueroa-Clarevega A. Bilder D. (2015). Malignant drosophila Tumors Interrupt Insulin Signaling to Induce Cachexia-like Wasting. Dev. Cel 33 (1), 47–55. 10.1016/j.devcel.2015.03.001 PMC4390765 25850672

[B71] Finlin B. S. Zhu B. Confides A. L. Westgate P. M. Harfmann B. D. Dupont-Versteegden E. E. (2017). Mast Cells Promote Seasonal white Adipose Beiging in Humans. Diabetes 66 (5), 1237–1246. 10.2337/db16-1057 28250021 PMC5399616

[B163] Freychet P. (1990). Pancreatic hormones. Hormones: from molecules to disease. Editors Baulieu E.-E. Kelly P. A. (Chapman and Hall: New York.

[B72] Fu M. Xu L. Chen X. Han W. Ruan C. Li J. (2019). Neural Crest Cells Differentiate into Brown Adipocytes and Contribute to Periaortic Arch Adipose Tissue Formation. Atvb 39 (8), 1629–1644. 10.1161/atvbaha.119.312838 31189430

[B73] Funcke J.-B. Scherer P. E. (2019). Beyond Adiponectin and Leptin: Adipose Tissue-Derived Mediators of Inter-organ Communication. J. Lipid Res. 60, 1648–1697. 10.1194/jlr.r094060 31209153 PMC6795086

[B74] Gáliková M. Diesner M. Klepsatel P. Hehlert P. Xu Y. Bickmeyer I. (2015). Energy Homeostasis Control in drosophila Adipokinetic Hormone Mutants. Genetics 201 (2), 665–683. 10.1534/genetics.115.178897 26275422 PMC4596676

[B75] Garrido D. Rubin T. Poidevin M. Maroni B. Le Rouzic A. Parvy J. P. (2015). Fatty Acid Synthase Cooperates with Glyoxalase 1 to Protect against Sugar Toxicity. Plos Genet. 11 (2), 1–26. 10.1371/journal.pgen.1004995 PMC4334898 25692475

[B76] Gastaldelli A. (2011). Role of Beta-Cell Dysfunction, Ectopic Fat Accumulation and Insulin Resistance in the Pathogenesis of Type 2 Diabetes Mellitus. Diabetes Res. Clin. Pract. 93 Suppl 1 (Suppl. 1), S60–S65. 10.1016/S0168-8227(11)70015-8 21864753

[B77] Géminard C. Rulifson E. J. Léopold P. (2009). Remote Control of Insulin Secretion by Fat Cells in Drosophila. Cel Metab 10 (3), 199–207. 10.1016/j.cmet.2009.08.002 19723496

[B78] Gesta S. Tseng Y.-H. Kahn C. R. (2007). Developmental Origin of Fat: Tracking Obesity to its Source. Cell 131, 242–256. 10.1016/j.cell.2007.10.004 17956727

[B79] Ghosh A. C. O’Connor M. B. (2014). Systemic Activin Signaling Independently Regulates Sugar Homeostasis, Cellular Metabolism, and pH Balance in *Drosophila melanogaster* . Proc. Natl. Acad. Sci. U S A. 111 (15), 5729–5734. 10.1073/pnas.1319116111 24706779 PMC3992655

[B80] Ghosh A. C. Tattikota S. G. Liu Y. Comjean A. Hu Y. Barrera V. (2020). Drosophila Pdgf/vegf Signaling from Muscles to Hepatocyte-like Cells Protects against Obesity. Elife 9, 1–61. 10.7554/eLife.56969 PMC7752135 33107824

[B81] Giannakou M. E. Goss M. Jünger M. A. Hafen E. Leevers S. J. Partridge L. (2004). Long-lived Drosophila with Over-expressed dFOXO in Adult Fat Body. Science 305 (5682), 361. 10.1126/science.1098219 15192154

[B82] Goldberg A. A. Bourque S. D. Kyryakov P. Boukh-Viner T. Gregg C. Beach A. (2009). A Novel Function of Lipid Droplets in Regulating Longevity. Biochem. Soc. Trans., 1050. 10.1042/bst0371050 19754450

[B83] Grauer W. O. Moss A. A. Cann C. E. Goldberg H. I. (1984). Quantification of Body Fat Distribution in the Abdomen Using Computed Tomography. Am. J. Clin. Nutr. 39 (4), 631–637. 10.1093/ajcn/39.4.631 6711470

[B84] Grönke S. Beller M. Fellert S. Ramakrishnan H. Jäckle H. Kühnlein R. P. (2003). Control of Fat Storage by a Drosophila PAT Domain Protein. Curr. Biol. 13 (7), 603–606. 10.1016/s0960-9822(03)00175-1 12676093

[B85] Grönke S. Clarke D. F. Broughton S. Andrews T. D. Partridge L. (2010). Molecular Evolution and Functional Characterization of Drosophila Insulin-like Peptides. Plos Genet. 6 (2). 10.1371/journal.pgen.1000857 PMC2829060 20195512

[B86] Grönke S. Mildner A. Fellert S. Tennagels N. Petry S. Müller G. (2005). Brummer Lipase Is an Evolutionary Conserved Fat Storage Regulator in Drosophila. Cel Metab 1 (5), 323–330. 10.1016/j.cmet.2005.04.003 16054079

[B87] Guarner A. Morris R. Korenjak M. Boukhali M. Zappia M. P. Van Rechem C. (2017). E2F/DP Prevents Cell-Cycle Progression in Endocycling Fat Body Cells by Suppressing dATM Expression. Dev. Cel 43 (6), 689–703. 10.1016/j.devcel.2017.11.008 PMC5901703 29233476

[B88] Guerra C. Koza R. A. Yamashita H. Walsh K. Kozak L. P. (1998). Emergence of Brown Adipocytes in white Fat in Mice Is under Genetic Control. Effects on Body Weight and Adiposity. J. Clin. Invest. 102 (2), 412–420. 10.1172/jci3155 9664083 PMC508900

[B89] Guilherme A. Virbasius J. V. Puri V. Czech M. P. (2008). Adipocyte Dysfunctions Linking Obesity to Insulin Resistance and Type 2 Diabetes. Nat. Rev. Mol. Cel Biol 9, 367–377. 10.1038/nrm2391 PMC2886982 18401346

[B90] Gupta R. K. (2014). AdipocytesCurrent Biology. Curr. Biol. Vol. 24, R988. 10.1016/j.cub.2014.09.003 25442852

[B91] Gutierrez E. Wiggins D. Fielding B. Gould A. P. (2007). Specialized Hepatocyte-like Cells Regulate Drosophila Lipid Metabolism. Nature 445 (7125), 275–280. 10.1038/nature05382 17136098

[B92] Harms M. Seale P. (2013). Brown and Beige Fat: Development, Function and Therapeutic Potential. Nat. Med., Vol. 19, 1252–1263. 10.1038/nm.3361 24100998

[B93] Hartenstein V. Jan Y. N. (1992). Studying Drosophila Embryogenesis with P-lacZ Enhancer Trap Lines. Roux's Arch. Dev. Biol. 201 (4), 194–220. 10.1007/bf00188752 28305845

[B94] Hasygar K. Deniz O. Liu Y. Gullmets J. Hynynen R. Ruhanen H. (2021). Coordinated Control of Adiposity and Growth by Anti‐anabolic Kinase ERK7. EMBO Rep. 22 (2), 1–16. 10.15252/embr.201949602 PMC7857433 33369866

[B95] Haunerland N. H. Shirk P. D. (1995). Regional and Functional Differentiation in the Insect Fat Body. Annu. Rev. Entomol. 40 (1), 121–145. 10.1146/annurev.en.40.010195.001005

[B96] Havula E. Hietakangas V. (2012). Glucose Sensing by ChREBP/MondoA-Mlx Transcription Factors. Vol. 23, Seminars in Cell and Developmental Biology. Semin. Cel Dev Biol, 640–647. 10.1016/j.semcdb.2012.02.007 22406740

[B97] Havula E. Teesalu M. Hyötyläinen T. Seppälä H. Hasygar K. Auvinen P. (2013). Mondo/ChREBP-Mlx-Regulated Transcriptional Network Is Essential for Dietary Sugar Tolerance in Drosophila. Plos Genet. 9 (4). 10.1371/journal.pgen.1003438 PMC3616910 23593032

[B98] Heine M. Fischer A. W. Schlein C. Jung C. Straub L. G. Gottschling K. (2018). Lipolysis Triggers a Systemic Insulin Response Essential for Efficient Energy Replenishment of Activated Brown Adipose Tissue in Mice. Cel Metab 28 (4), 644–655. 10.1016/j.cmet.2018.06.020 30033199

[B99] Hiraike Y. Waki H. Miyake K. Wada T. Oguchi M. Saito K. (2020). NFIA Differentially Controls Adipogenic and Myogenic Gene Program through Distinct Pathways to Ensure Brown and Beige Adipocyte Differentiation. Plos Genet. 16 (9), e1009044. 10.1371/journal.pgen.1009044 32991581 PMC7546476

[B100] Hiraike Y. Waki H. Yu J. Nakamura M. Miyake K. Nagano G. (2017). NFIA Co-localizes with PPARγ and Transcriptionally Controls the Brown Fat Gene Program. Nat. Cel Biol 19 (9), 1081–1092. 10.1038/ncb3590 PMC5885759 28812581

[B101] Hoang A. C. Yu H. Röszer T. (2021). Transcriptional Landscaping Identifies a Beige Adipocyte Depot in the Newborn Mouse. Cells 10 (9). 10.3390/cells10092368 PMC8470180 34572017

[B102] Hoffmann J. Romey R. Fink C. Yong L. Roeder T. (2013). Overexpression of Sir2 in the Adult Fat Body Is Sufficient to Extend Lifespan of Male and Female Drosophila. Aging (Albany NY) 5 (4), 315–327. 10.18632/aging.100553 23765091 PMC3651523

[B103] Holz A. Meise M. Janning W. (1997). Adepithelial Cells in *Drosophila melanogaster*: Origin and Cell Lineage. Mech. Dev. 62 (1), 93–101. 10.1016/s0925-4773(97)00654-0 9106170

[B104] Honegger B. Galic M. Köhler K. Wittwer F. Brogiolo W. Hafen E. (2008). Imp-L2, a Putative Homolog of Vertebrate IGF-Binding Protein 7, Counteracts Insulin Signaling in Drosophila and Is Essential for Starvation Resistance. J. Biol. 7 (3), 172. 10.1186/jbiol72 PMC2323038 18412985

[B105] Hoshizaki D. K. (1994). Krüppel Expression during Postembryonic Development of Drosophila. Dev. Biol. 163 (1), 133–140. 10.1006/dbio.1994.1129 8174769

[B106] Hoshizaki D. K. Blackburn T. Price C. Ghosh M. Miles K. Ragucci M. (1994). Embryonic Fat-Cell Lineage in *Drosophila melanogaster* . Development 120 (9), 2489–2499. 10.1242/dev.120.9.2489 7956826

[B107] Hoshizaki D. K. Lunz R. Johnson W. Ghosh M. (1995). Identification of Fat-Cell Enhancer Activity inDrosophila Melanogasterusing P-Element Enhancer Traps. Genome 38 (3), 497–506. 10.1139/g95-065 7557362

[B108] Hotamisligil G. S. (2017). Inflammation, Metaflammation and Immunometabolic Disorders. Nature 542, 177–185. 10.1038/nature21363 28179656

[B109] Hughson B. N. Shimell M. J. O’Connor M. B. (2021). AKH Signaling in *D. melanogaster* Alters Larval Development in a Nutrient-dependent Manner that Influences Adult Metabolism. Front. Physiol., 12. 10.3389/fphys.2021.619219 PMC7940354 33708137

[B110] Hyun S. Lee J. H. Jin H. Nam J. W. Namkoong B. Lee G. (2009). Conserved MicroRNA miR-8/miR-200 and its Target USH/FOG2 Control Growth by Regulating PI3K. Cell 139 (6), 1096–1108. 10.1016/j.cell.2009.11.020 20005803

[B111] Iijima K. Zhao L. Shenton C. Iijima-Ando K. (2009). Regulation of Energy Stores and Feeding by Neuronal and Peripheral CREB Activity in Drosophila. PLoS One 4 (12). 10.1371/journal.pone.0008498 PMC2795867 20041126

[B247] Ikeda K. Maretich P. Kajimura S. (2018). The Common and Distinct Features of Brown and Beige Adipocytes. Trends Endocrinol Metab. 29 (3), 191–200. 29366777 10.1016/j.tem.2018.01.001 PMC5826798

[B112] Imrie D. Sadler K. C. (2010). White Adipose Tissue Development in Zebrafish Is Regulated by Both Developmental Time and Fish Size. Dev. Dyn. 239 (11), 3013–3023. 10.1002/dvdy.22443 20925116 PMC3016641

[B113] Ingaramo M. C. Sánchez J. A. Perrimon N. Dekanty A. (2020). Fat Body P53 Regulates Systemic Insulin Signaling and Autophagy under Nutrient Stress via Drosophila Upd2 Repression. Cel Rep 33 (4), 321. 10.1016/j.celrep.2020.108321 PMC9036415 33113367

[B114] Isabel G. Martin J. R. Chidami S. Veenstra J. A. Rosay P. (2005). AKH-producing Neuroendocrine Cell Ablation Decreases Trehalose and Induces Behavioral Changes in Drosophila. Am. J. Physiol. - Regul. Integr. Comp. Physiol. 288 (2 57-2). 2004. 10.1152/ajpregu.00158.2004 15374818

[B115] Jin H. Narry Kim V. Hyun S. (2012). Conserved microRNA miR-8 Controls Body Size in Response to Steroid Signaling in Drosophila. Genes Dev. 26 (13), 1427–1432. 10.1101/gad.192872.112 22751499 PMC3403011

[B116] Johnson M. B. Butterworth F. M. (1985). Maturation and Aging of Adult Fat Body and Oenocytes in Drosophila as Revealed by Light Microscopic Morphometry. J. Morphol. 184 (1), 51–59. 10.1002/jmor.1051840106 3921720

[B117] Jung S. M. Sanchez-Gurmaches J. Guertin D. A. (2019). Brown Adipose Tissue Development and Metabolism. Handb Exp. Pharmacol. 251, 3–36. 10.1007/164_2018_168 30203328 PMC7330484

[B118] Kadereit B. Kumar P. Wang W.-J. Miranda D. Snapp E. L. Severina N. (2008). Evolutionarily Conserved Gene Family Important for Fat Storage. Proc. Natl. Acad. Sci. 105 (1), 94–99. 10.1073/pnas.0708579105 18160536 PMC2224239

[B119] Katheder N. S. Khezri R. O’Farrell F. Schultz S. W. Jain A. Schink M. K. O. (2017). Microenvironmental Autophagy Promotes Tumour Growth. Nature 541 (7637), 417–420. 10.1038/nature20815 28077876 PMC5612666

[B120] Khezri R. Holland P. Schoborg T. A. Abramovich I. Takáts S. Dillard C. (2021). Host Autophagy Mediates Organ Wasting and Nutrient Mobilization for Tumor Growth. EMBO J. 40 (18), e107336. 10.15252/embj.2020107336 34309071 PMC8441431

[B121] Kim S. K. Rulifson E. J. (20041670). Conserved Mechanisms of Glucose Sensing and Regulation by Drosophila Corpora Cardiaca Cells. Nature 431, 431316–431320. 10.1038/nature02897 15372035

[B122] Kir S. White J. P. Kleiner S. Kazak L. Cohen P. Baracos V. E. (2014). Tumour-derived PTH-Related Protein Triggers Adipose Tissue browning and Cancer Cachexia. Nature 513 (7516), 100–104. 10.1038/nature13528 25043053 PMC4224962

[B123] Kopecký J. Hodný Z. Rossmeisl M. Syrový I. Kozak L. P. (1996). Reduction of Dietary Obesity in aP2-Ucp Transgenic Mice: Physiology and Adipose Tissue Distribution. Am. J. Physiol. - Endocrinol. Metab. 270 (5 33-5). 10.1152/ajpendo.1996.270.5.E768 8967464

[B124] Koyama T. Mirth C. K. (2016). Growth-Blocking Peptides as Nutrition-Sensitive Signals for Insulin Secretion and Body Size Regulation. Plos Biol. 14 (2). 10.1371/journal.pbio.1002392 PMC4771208 26928023

[B125] Krahmer N. Hilger M. Kory N. Wilfling F. Stoehr G. Mann M. (2013). Protein Correlation Profiles Identify Lipid Droplet Proteins with High Confidence. Mol. Cel Proteomics 12 (5), 1115–1126. 10.1074/mcp.m112.020230 PMC3650325 23319140

[B126] Kwon H. J. Waghmare I. Verghese S. Singh A. Singh A. Kango-Singh M. (2015). Drosophila C-Terminal Src Kinase Regulates Growth via the Hippo Signaling Pathway. Dev. Biol. 397 (1), 67–76. 10.1016/j.ydbio.2014.10.010 25446534

[B127] Lamers D. Famulla S. Wronkowitz N. Hartwig S. Lehr S. Ouwens D. M. (2011). Dipeptidyl Peptidase 4 Is a Novel Adipokine Potentially Linking Obesity to the Metabolic Syndrome. Diabetes 60 (7), 1917–1925. 10.2337/db10-1707 21593202 PMC3121429

[B128] Larsen W. J. (1976). Cell Remodeling in the Fat Body of an Insect. Tissue Cell 8 (1), 73–92. 10.1016/0040-8166(76)90021-5 178069

[B129] Lawrence P. A. Johnston P. (1986). Observations on Cell Lineage of Internal Organs of Drosophila. J. Embryol. Exp. Morphol. 91, 251–266. 10.1242/dev.91.1.251 3711788

[B130] Lee B. Barretto E. C. Grewal S. S. (2019). TORC1 Modulation in Adipose Tissue Is Required for Organismal Adaptation to Hypoxia in Drosophila. Nat. Commun. 10 (1). 10.1038/s41467-019-09643-7 PMC6478872 31015407

[B131] Lee G. J. Han G. Yun H. M. Lim J. J. Noh S. Lee J. (2018). Steroid Signaling Mediates Nutritional Regulation of Juvenile Body Growth via IGF-Binding Protein in Drosophila. Proc. Natl. Acad. Sci. U S A. 115 (23), 5992–5997. 10.1073/pnas.1718834115 29784791 PMC6003328

[B132] Lee G. J. Jun J. W. Hyun S. (2015). MicroRNA miR-8 Regulates Multiple Growth Factor Hormones Produced from Drosophila Fat Cells. Insect Mol. Biol. 24 (3), 311–318. 10.1111/imb.12156 25492518

[B133] Lee G. Park J. H. (2004). Hemolymph Sugar Homeostasis and Starvation-Induced Hyperactivity Affected by Genetic Manipulations of the Adipokinetic Hormone-Encoding Gene in *Drosophila melanogaster* . Genetics 167 (1), 311–323. 10.1534/genetics.167.1.311 15166157 PMC1470856

[B134] Lee K.-S. You K.-H. Choo J.-K. Han Y.-M. Yu K. (2004). Drosophila Short Neuropeptide F Regulates Food Intake and Body Size. J. Biol. Chem. 279 (49), 50781–50789. 10.1074/jbc.m407842200 15385546

[B135] Lehr S. Hartwig S. Lamers D. Famulla S. Müller S. Hanisch F. G. (2012). Identification and Validation of Novel Adipokines Released from Primary Human Adipocytes. Mol. Cel Proteomics 11 (1), M111–M010504. 10.1074/mcp.M111.010504 PMC3270100 21947364

[B136] Lehr S. Hartwig S. Sell H. (2012). Adipokines: A Treasure Trove for the Discovery of Biomarkers for Metabolic Disorders. Prot. Clin. Appl. 6, 91–101. 10.1002/prca.201100052 22213627

[B137] Li S. Yu X. Feng Q. (2019). Fat Body Biology in the Last Decade. Annu. Rev. Entomol. 64, 315–333. 10.1146/annurev-ento-011118-112007 30312553

[B138] Lidell M. E. Betz M. J. Leinhard O. D. Heglind M. Elander L. Slawik M. (2013). Evidence for Two Types of Brown Adipose Tissue in Humans. Nat. Med. 19 (5), 631–634. 10.1038/nm.3017 23603813

[B139] Lodge W. Zavortink M. Golenkina S. Froldi F. Dark C. Cheung S. (2021). Tumor-derived MMPs Regulate Cachexia in a Drosophila Cancer Model. Dev. Cel 56 (18), 2664–2680. 10.1016/j.devcel.2021.08.008 34473940

[B140] Loft A. Forss I. Siersbæk M. S. Schmidt S. F. Larsen A. S. B. Madsen J. G. S. (2015). Browning of Human Adipocytes Requires KLF11 and Reprogramming of PPARγ Superenhancers. Genes Dev. 29 (1), 7–22. 10.1101/gad.250829.114 25504365 PMC4281566

[B141] Lowell B. B. S-Susulic V. Hamann A. Lawitts J. A. Himms-Hagen J. Boyer B. B. (1993). Development of Obesity in Transgenic Mice after Genetic Ablation of Brown Adipose Tissue. Nature 366 (6457), 740–742. 10.1038/366740a0 8264795

[B142] Lshibashi J. Seale P. (2010). Beige Can Be Slimming. Sci. Sci. Vol. 328, 1113–1114. 10.1126/science.1190816 PMC2907667 20448151

[B143] Luong N. Davies C. R. Wessells R. J. Graham S. M. King M. T. Veech R. (2006). Activated FOXO-Mediated Insulin Resistance Is Blocked by Reduction of TOR Activity. Cel Metab 4 (2), 133–142. 10.1016/j.cmet.2006.05.013 16890541

[B144] Martínez B. A. Hoyle R. G. Yeudall S. Granade M. E. Harris T. E. David Castle J. (2020). Innate Immune Signaling in Drosophila Shifts Anabolic Lipid Metabolism from Triglyceride Storage to Phospholipid Synthesis to Support Immune Function. Plos Genet. (11), 16. 10.1371/journal.pgen.1009192 PMC7721134 33227003

[B145] Mattila J. Havula E. Suominen E. Teesalu M. Surakka I. Hynynen R. (2015). Mondo-Mlx Mediates Organismal Sugar Sensing through the Gli-Similar Transcription Factor Sugarbabe. Cel Rep 13 (2), 350–364. 10.1016/j.celrep.2015.08.081 26440885

[B146] Mattila J. Hietakangas V. (2017). Regulation of Carbohydrate Energy Metabolism in *Drosophila melanogaster* . Genetics 207 (4), 1231–1253. 10.1534/genetics.117.199885 29203701 PMC5714444

[B147] Meschi E. Léopold P. Delanoue R. (2019). An EGF-Responsive Neural Circuit Couples Insulin Secretion with Nutrition in Drosophila. Dev. Cel 48 (1), 76–86. 10.1016/j.devcel.2018.11.029 30555002

[B148] Miller J. M. Oligino T. Pazdera M. López A. J. Hoshizaki D. K. (2002). Identification of Fat-Cell Enhancer Regions inDrosophila Melanogaster. Insect Mol. Biol. 11 (1), 67–77. 10.1046/j.0962-1075.2001.00310.x 11841504

[B149] Mirth C. K. Riddiford L. M. (2007). Size Assessment and Growth Control: How Adult Size Is Determined in Insects. BioEssays Vol. 29, 344–355. 10.1002/bies.20552 17373657

[B150] Molaei M. Vandehoef C. Karpac J. (2019). NF-κB Shapes Metabolic Adaptation by Attenuating Foxo-Mediated Lipolysis in Drosophila. Dev. Cel 49 (5), 802–810.e6. 10.1016/j.devcel.2019.04.009 PMC6548632 31080057

[B151] Moore L. A. Broihier H. T. Van Doren M. Lehmann R. (1998). Gonadal Mesoderm and Fat Body Initially Follow a Common Developmental Path in Drosophila. Development 125 (5), 837–844. 10.1242/dev.125.5.837 9449666

[B152] Müller H. (1968). Fine Structure and Lipid Formation in Fat Cells of the Perimeningeal Tissue of Lampreys under normal and Experimental Conditions. Z. Zellforsch Mikrosk Anat. 84 (4), 585–608. 5700565

[B153] Musselman L. P. Fink J. L. Ramachandran P. V. Patterson B. W. Okunade A. L. Maier E. (2013). Role of Fat Body Lipogenesis in protection against the Effects of Caloric Overload in drosophila. J. Biol. Chem. 288 (12), 8028–8042. 10.1074/jbc.m112.371047 23355467 PMC3605622

[B154] Musselman L. P. Kühnlein R. P. (2018). Drosophila as a Model to Study Obesity and Metabolic Disease. Vol. 121. J. Exp. BiologyJ Exp Biol. 10.1242/jeb.163881 29514880

[B155] Na J. Musselman L. P. Pendse J. Baranski T. J. Bodmer R. Ocorr K. (2013). A Drosophila Model of High Sugar Diet-Induced Cardiomyopathy. Plos Genet. 9 (1), e1003175. 10.1371/journal.pgen.1003175 23326243 PMC3542070

[B156] Nässel D. R. Wegener C. (2011). A Comparative Review of Short and Long Neuropeptide F Signaling in Invertebrates: Any Similarities to Vertebrate Neuropeptide Y Signaling. Peptides 32, 1335–1355. 10.1016/j.peptides.2011.03.013 21440021

[B157] Neugnot V. Moulin G. Dubreucq E. Bigey F. (2002). The Lipase/acyltransferase fromCandida Parapsilosis. Eur. J. Biochem. 269 (6), 1734–1745. 10.1046/j.1432-1327.2002.02828.x 11895444

[B158] Ng M. Fleming T. Robinson M. Thomson B. Graetz N. Margono C. (2014). Global, Regional, and National Prevalence of Overweight and Obesity in Children and Adults during 1980-2013: A Systematic Analysis for the Global Burden of Disease Study 2013. Lancet 384 (9945), 766–781. 10.1016/S0140-6736(14)60460-8 24880830 PMC4624264

[B159] Novak M. Monkus E. Pardo V. (1971). Human Neonatal Subcutaneous Adipose Tissue. Function and Ultrastructure. Biol. Neonate 19 (4–6), 306–321. 10.1159/000240425 5149287

[B160] Okamoto N. Yamanaka N. Yagi Y. Nishida Y. Kataoka H. O’Connor M. B. (2009). A Fat Body-Derived IGF-like Peptide Regulates Postfeeding Growth in Drosophila. Dev. Cel 17 (6), 885–891. 10.1016/j.devcel.2009.10.008 PMC4423396 20059957

[B161] Olofsson S.-O. Boström P. Andersson L. Rutberg M. Levin M. Perman J. (2008). Triglyceride Containing Lipid Droplets and Lipid Droplet-Associated Proteins. Curr. Opin. Lipidol. 19, 441–447. 10.1097/mol.0b013e32830dd09b 18769224

[B162] Ottaviani E. Malagoli D. Franceschi C. (2011). The Evolution of the Adipose Tissue: A Neglected enigma. Gen. Comp. Endocrinol. 174, 1–4. 10.1016/j.ygcen.2011.06.018 21781968

[B164] Palanker L. Tennessen J. M. Lam G. Thummel C. S. (2009). Drosophila HNF4 Regulates Lipid Mobilization and β-Oxidation. Cel Metab 9 (3), 228–239. 10.1016/j.cmet.2009.01.009 PMC2673486 19254568

[B165] Palanker Musselman L. Fink J. L. Narzinski K. Ramachandran P. V. Sukumar Hathiramani S. Cagan R. L. (2011). A High-Sugar Diet Produces Obesity and Insulin Resistance in Wild-type Drosophila. DMM Dis. Model. Mech. 4 (6), 842–849. 10.1242/dmm.007948 21719444 PMC3209653

[B166] Palm W. Sampaio J. L. Brankatschk M. Carvalho M. Mahmoud A. Shevchenko A. (2012). Lipoproteins in Drosophila Melanogaster-Aassembly, Function, and Influence on Tissue Lipid Composition. Plos Genet. 8 (7), e1002828. 10.1371/journal.pgen.1002828 22844248 PMC3406001

[B167] Palu R. A. S. Thummel C. S. (2016). Sir2 Acts through Hepatocyte Nuclear Factor 4 to Maintain Insulin Signaling and Metabolic Homeostasis in Drosophila. Plos Genet. 12 (4). 10.1371/journal.pgen.1005978 PMC4825955 27058248

[B168] Parisi F. Riccardo S. Zola S. Lora C. Grifoni D. Brown L. M. (2013). DMyc Expression in the Fat Body Affects DILP2 Release and Increases the Expression of the Fat Desaturase Desat1 Resulting in Organismal Growth. Dev. Biol. 379 (1), 64–75. 10.1016/j.ydbio.2013.04.008 23608455 PMC3712331

[B169] Parra-Peralbo E. Culi J. (2011). Drosophila Lipophorin Receptors Mediate the Uptake of Neutral Lipids in Oocytes and Imaginal Disc Cells by an Endocytosis-independent Mechanism. Plos Genet. 7 (2), e1001297. 10.1371/journal.pgen.1001297 21347279 PMC3037410

[B170] Pasco M. Y. Léopold P. (2012). High Sugar-Induced Insulin Resistance in Drosophila Relies on the Lipocalin Neural Lazarillo. PLoS One 7 (5), e36583. 10.1371/journal.pone.0036583 22567167 PMC3342234

[B171] Pathak H. Varghese J. (2021). Edem1 Activity in the Fat Body Regulates Insulin Signalling and Metabolic Homeostasis in Drosophila. Life Sci. Alliance 4 (8), 1079. 10.26508/lsa.202101079 PMC8321676 34140347

[B172] Petrovic N. Walden T. B. Shabalina I. G. Timmons J. A. Cannon B. Nedergaard J. (2010). Chronic Peroxisome Proliferator-Activated Receptor γ (PPARγ) Activation of Epididymally Derived White Adipocyte Cultures Reveals a Population of Thermogenically Competent, UCP1-Containing Adipocytes Molecularly Distinct from Classic Brown Adipocytes. J. Biol. Chem. 285 (10), 7153–7164. 10.1074/jbc.m109.053942 20028987 PMC2844165

[B173] Petruzzelli M. Wagner E. F. (2016). Mechanisms of Metabolic Dysfunction in Cancer-Associated Cachexia. Genes Dev. 30, 489–501. 10.1101/gad.276733.115 26944676 PMC4782044

[B174] Pospisilik J. A. Schramek D. Schnidar H. Cronin S. J. F. Nehme N. T. Zhang X. (2010). Drosophila Genome-wide Obesity Screen Reveals Hedgehog as a Determinant of Brown versus White Adipose Cell Fate. Cell 140 (1), 148–160. 10.1016/j.cell.2009.12.027 20074523

[B175] Post S. Karashchuk G. Wade J. D. Sajid W. De Meyts P. Tatar M. (2018). Drosophila Insulin-like Peptides DILP2 and DILP5 Differentially Stimulate Cell Signaling and Glycogen Phosphorylase to Regulate Longevity. Front. Endocrinol. (Lausanne) 9 (MAY). 245. 10.3389/fendo.2018.00245 29892262 PMC5985746

[B176] Rajan A. Perrimon N. (2012). Drosophila Cytokine Unpaired 2 Regulates Physiological Homeostasis by Remotely Controlling Insulin Secretion. Cell 151 (1), 123–137. 10.1016/j.cell.2012.08.019 23021220 PMC3475207

[B177] Reis T. van Gilst M. R. Hariharan I. K. (2010). A Buoyancy-Based Screen of drosophila Larvae for Fat- Storage Mutants Reveals a Role for Sir2 in Coupling Fat Storage to Nutrient Availability. Plos Genet. 6 (11), 206. 10.1371/journal.pgen.1001206 PMC2978688 21085633

[B178] Ren G. R. Hauser F. Rewitz K. F. Kondo S. Engelbrecht A. F. Didriksen A. K. (2015). CCHamide-2 Is an Orexigenic Brain-Gut Peptide in Drosophila. PLoS One 10 (7). 10.1371/journal.pone.0133017 PMC4500396 26168160

[B179] Riechmann V. Rehorn K. P. (1998). The Genetic Control of the Distinction between Fat Body and Gonadal Mesoderm in Drosophila. Development 125(4):713–723. 9435291 10.1242/dev.125.4.713

[B180] Rodríguez-Vázquez M. Vaquero D. Parra-Peralbo E. Mejía-Morales J. E. Culi J. (2015). Drosophila Lipophorin Receptors Recruit the Lipoprotein LTP to the Plasma Membrane to Mediate Lipid Uptake. Plos Genet. 11 (6). 10.1371/journal.pgen.1005356 PMC4486166 26121667

[B181] Rőszer T. Kiss-Tóth É. D. (2014). FMRF-amide Is a Glucose-Lowering Hormone in the Snail Helix Aspersa. Cell Tissue Res 358 (2), 371–383. 10.1007/s00441-014-1966-x 25096715

[B182] Roth S. W. Bitterman M. D. Birnbaum M. J. Bland M. L. (2018). Innate Immune Signaling in Drosophila Blocks Insulin Signaling by Uncoupling PI(3,4,5)P 3 Production and Akt Activation. Cel Rep 22 (10), 2550–2556. 10.1016/j.celrep.2018.02.033 PMC5866056 29514084

[B183] Rusten T. E. Lindmo K. Juhász G. Sass M. Seglen P. O. Brech A. (2004). Programmed Autophagy in the Drosophila Fat Body Is Induced by Ecdysone through Regulation of the PI3K Pathway. Dev. Cel 7 (2), 179–192. 10.1016/j.devcel.2004.07.005 15296715

[B184] Rydén M. Arner P. (2007). Fat Loss in Cachexia-Is There a Role for Adipocyte Lipolysis? Clin. Nutr. Vol. 26, 1–6. 10.1016/j.clnu.2006.09.009 17095126

[B185] Saavedra P. Perrimon N. (2019). Drosophila as a Model for Tumor-Induced Organ Wasting. Adv. Exp. Med. Biol., 191–205. 10.1007/978-3-030-23629-8_11 31520356

[B186] Saltiel A. R. (2012). Insulin Resistance in the Defense against Obesity. Cel Metab. 15, 798–804. 10.1016/j.cmet.2012.03.001 22682220

[B187] Saltiel A. R. Kahn C. R. (2001). Insulin Signalling and the Regulation of Glucose and Lipid Metabolism. Nature 414, 799–806. 10.1038/414799a 11742412

[B188] Sam S. Leise W. Keiko Hoshizaki D. (1996). The Serpent Gene Is Necessary for Progression through the Early Stages of Fat-Body Development. Mech. Dev. 60 (2), 197–205. 10.1016/s0925-4773(96)00615-6 9025072

[B189] Sano H. Nakamura A. Texada M. J. Truman J. W. Ishimoto H. Kamikouchi A. (2015). The Nutrient-Responsive Hormone CCHamide-2 Controls Growth by Regulating Insulin-like Peptides in the Brain of *Drosophila melanogaster* . Plos Genet. 11 (5). 10.1371/journal.pgen.1005209 PMC4447355 26020940

[B190] Sato M. Kornberg T. B. (2002). FGF Is an Essential Mitogen and Chemoattractant for the Air Sacs of the Drosophila Tracheal System. Dev. Cel 3 (2), 195–207. 10.1016/s1534-5807(02)00202-2 12194851

[B191] Saxton R. A. Sabatini D. M. (2017). mTOR Signaling in Growth, Metabolism, and Disease. Cell Vol. 168, 960–976. 10.1016/j.cell.2017.02.004 PMC5394987 28283069

[B192] Schaffer M. H. Noyes B. E. Slaughter C. A. Thorne G. C. Gaskell S. J. (1990). The Fruitfly *Drosophila melanogaster* Contains a Novel Charged Adipokinetic-Hormone-Family Peptide. Biochem. J. 269 (2), 315–320. 10.1042/bj2690315 2117437 PMC1131578

[B193] Schwartz D. R. Lazar M. A. (2011). Human Resistin: Found in Translation from Mouse to Man. Trends Endocrinol. Metab. 22, 259–265. 10.1016/j.tem.2011.03.005 21497511 PMC3130099

[B194] Scopelliti A. Bauer C. Yu Y. Zhang T. Kruspig B. Murphy D. J. (2019). A Neuronal Relay Mediates a Nutrient Responsive Gut/Fat Body Axis Regulating Energy Homeostasis in Adult Drosophila. Cel Metab 29 (2), 269–284. 10.1016/j.cmet.2018.09.021 PMC6370946 30344016

[B195] Scott R. C. Schuldiner O. Neufeld T. P. (2004). Role and Regulation of Starvation-Induced Autophagy in the Drosophila Fat Body. Dev. Cel 7 (2), 167–178. 10.1016/j.devcel.2004.07.009 15296714

[B196] Seale P. Bjork B. Yang W. Kajimura S. Chin S. Kuang S. (2008). PRDM16 Controls a Brown Fat/skeletal Muscle Switch. Nature 454 (7207), 961–967. 10.1038/nature07182 18719582 PMC2583329

[B197] Seale P. Conroe H. M. Estall J. Kajimura S. Frontini A. Ishibashi J. (2011). Prdm16 Determines the Thermogenic Program of Subcutaneous white Adipose Tissue in Mice. J. Clin. Invest. 121 (1), 96–105. 10.1172/jci44271 21123942 PMC3007155

[B198] Semaniuk U. Piskovatska V. Strilbytska O. Strutynska T. Burdyliuk N. Vaiserman A. (2021). Drosophila Insulin-like Peptides: From Expression to Functions – a Review Entomologia Experimentalis et Applicata (John Wiley & Sons), Vol. 169, 195–208. 10.1111/eea.12981

[B199] Semenza G. L. (2014). Oxygen Sensing, Hypoxia-Inducible Factors, and Disease Pathophysiology. Annu. Rev. Pathol. Mech. Dis. 9, 47–71. 10.1146/annurev-pathol-012513-104720 23937437

[B200] Sharp L. Z. Shinoda K. Ohno H. Scheel D. W. Tomoda E. Ruiz L. (2012). Human BAT Possesses Molecular Signatures that Resemble Beige/Brite Cells. PLoS One 7 (11), e49452. 10.1371/journal.pone.0049452 23166672 PMC3500293

[B201] Slaidina M. Delanoue R. Gronke S. Partridge L. Léopold P. (2009). A Drosophila Insulin-like Peptide Promotes Growth during Nonfeeding States. Dev. Cel 17 (6), 874–884. 10.1016/j.devcel.2009.10.009 PMC2806523 20059956

[B202] Snijder M. B. Dekker J. M. Visser M. Bouter L. M. Stehouwer C. D. Kostense P. J. (2003). Associations of Hip and Thigh Circumferences Independent of Waist Circumference with the Incidence of Type 2 Diabetes: The Hoorn Study. Am. J. Clin. Nutr. 77 (5), 1192–1197. 10.1093/ajcn/77.5.1192 12716671

[B203] Snijder M. B. Dekker J. M. Visser M. Yudkin J. S. Stehouwer C. D. A. Bouter L. M. (2003). Larger Thigh and Hip Circumferences Are Associated with Better Glucose Tolerance: The Hoorn Study. Obes. Res. 11 (1), 104–111. 10.1038/oby.2003.18 12529492

[B204] Song A. Dai W. Jang M. J. Medrano L. Li Z. Zhao H. (2020). Low- and High-Thermogenic Brown Adipocyte Subpopulations Coexist in Murine Adipose Tissue. J. Clin. Invest. 130 (1), 247–257. 10.1172/JCI129167 31573981 PMC6934193

[B205] Song W. Cheng D. Hong S. Sappe B. Hu Y. Wei N. (2017). Midgut-Derived Activin Regulates Glucagon-like Action in the Fat Body and Glycemic Control. Cel Metab 25 (2), 386–399. 10.1016/j.cmet.2017.01.002 PMC5373560 28178568

[B206] Song W. Kir S. Hong S. Hu Y. Wang X. Binari R. (2019). Tumor-Derived Ligands Trigger Tumor Growth and Host Wasting via Differential MEK Activation. Dev. Cel 48 (2), 277–286. 10.1016/j.devcel.2018.12.003 PMC6368352 30639055

[B207] Sousa-Nunes R. Yee L. L. Gould A. P. (2011). Fat Cells Reactivate Quiescent Neuroblasts via TOR and Glial Insulin Relays in Drosophila. Nature 471 (7339), 508–513. 10.1038/nature09867 21346761 PMC3146047

[B208] Stephens J. M. (2012). The Fat Controller: Adipocyte Development. Plos Biol. 10 (11), e1001436. 10.1371/journal.pbio.1001436 23209380 PMC3507952

[B209] Suzawa M. Muhammad N. M. Joseph B. S. Bland M. L. (2019). The Toll Signaling Pathway Targets the Insulin-like Peptide Dilp6 to Inhibit Growth in Drosophila. Cel Rep 28 (6), 1439–1446. 10.1016/j.celrep.2019.07.015 31390559

[B210] Tan B. K. Adya R. Randeva H. S. (2010). Omentin: A Novel Link between Inflammation, Diabesity, and Cardiovascular Disease. Trends Cardiovasc. Med. 20, 143–148. 10.1016/j.tcm.2010.12.002 21742269

[B211] Tchkonia T. Tchoukalova Y. D. Giorgadze N. Pirtskhalava T. Karagiannides I. Forse R. A. (2005). Abundance of Two Human Preadipocyte Subtypes with Distinct Capacities for Replication, Adipogenesis, and Apoptosis Varies Among Fat Depots. Am. J. Physiol. Endocrinol. Metab. 288 (1 51-1), E267–E277. 10.1152/ajpendo.00265.2004 15383371

[B212] Teixeira L. Rabouille C. Rørth P. Ephrussi A. Vanzo N. F. (2003). Drosophila Perilipin/ADRP Homologue Lsd2 Regulates Lipid Metabolism. Mech. Dev. 120 (9), 1071–1081. 10.1016/s0925-4773(03)00158-8 14550535

[B213] Terhzaz S. Cabrero P. Brinzer R. A. Halberg K. A. Dow J. A. T. Davies S. A. (2015). A novel role of Drosophila cytochrome P450-4e3 in permethrin insecticide tolerance. Insect Biochem. Mol. Biol. 67, 38–46. 10.1016/j.ibmb.2015.06.002 26073628 PMC4673087

[B214] Texada M. J. Jørgensen A. F. Christensen C. F. Koyama T. Malita A. Smith D. K. (2019). A Fat-Tissue Sensor Couples Growth to Oxygen Availability by Remotely Controlling Insulin Secretion. Nat. Commun. 10 (1), 1–16. 10.1038/s41467-019-09943-y 31028268 PMC6486587

[B215] The mesoderm and its derivatives (1993). in The Development of Drosophila melanogasterCold Spring Harbor. Editors BM A. (New York: Cold Spring Harbor Laboratory Press).

[B216] Thiam A. R. Beller M. (2017). The Why, when and How of Lipid Droplet Diversity, J. Cel Sci, 130. 315–324. 10.1242/jcs.192021 28049719

[B217] TM R. (1978). “Fat Body,” in The Genetics and Biology of Drosophila. Editors Ashburner M. Wright T. R. F. (London: Academic Press), 561–601.

[B218] Todorčević M. Kjær M. A. Djaković N. Vegusdal A. Torstensen B. E. Ruyter B. (2009). N-3 HUFAs Affect Fat Deposition, Susceptibility to Oxidative Stress, and Apoptosis in Atlantic salmon Visceral Adipose Tissue. Comp. Biochem. Physiol. - B Biochem. Mol. Biol. 152 (2), 135–143. 19010438 10.1016/j.cbpb.2008.10.009

[B219] Tsoli M. Swarbrick M. M. Robertson G. R. (2016). Lipolytic and Thermogenic Depletion of Adipose Tissue in Cancer Cachexia. Semin. Cel Dev. Biol. 54, 68–81. 10.1016/j.semcdb.2015.10.039 26529279

[B220] Ugrankar R. Berglund E. Akdemir F. Tran C. Kim M. S. Noh J. (2015). Drosophila Glucome Screening Identifies Ck1alpha as a Regulator of Mammalian Glucose Metabolism. Nat. Commun. 6, 7102. 10.1038/ncomms8102 25994086 PMC4455130

[B221] Ugrankar R. Bowerman J. Hariri H. Chandra M. Chen K. Bossanyi M. F. (2019). Drosophila Snazarus Regulates a Lipid Droplet Population at Plasma Membrane-Droplet Contacts in Adipocytes. Dev. Cel 50 (5), 557–572. 10.1016/j.devcel.2019.07.021 PMC7446143 31422916

[B222] Ugrankar R. Liu Y. Provaznik J. Schmitt S. Lehmann M. (2011). Lipin Is a Central Regulator of Adipose Tissue Development and Function in *Drosophila melanogaster* . Mol. Cel Biol 31 (8), 1646–1656. 10.1128/mcb.01335-10 PMC3126333 21300783

[B223] Ussar S. Lee K. Y. Dankel S. N. Boucher J. Haering M. F. Kleinridders A. (2014). ASC-1, PAT2, and P2RX5 Are Cell Surface Markers for white, Beige, and Brown Adipocytes. Sci. Transl Med. 6 (247), 247ra103. 10.1126/scitranslmed.3008490 PMC4356008 25080478

[B224] Waldén T. B. Hansen I. R. Timmons J. A. Cannon B. Nedergaard J. (2012). Recruited vs. Nonrecruited Molecular Signatures of Brown, "brite," and white Adipose Tissues. Am. J. Physiol. Endocrinol. Metab. 302 (1), E19–E31. 10.1152/ajpendo.00249.2011 21828341

[B225] Walther T. C. Farese R. V. (2012). Lipid Droplets and Cellular Lipid Metabolism. Annu. Rev. Biochem. 81, 687–714. 10.1146/annurev-biochem-061009-102430 22524315 PMC3767414

[B226] Wang B. Moya N. Niessen S. Hoover H. Mihaylova M. M. Shaw R. J. (2011). A Hormone-dependent Module Regulating Energy Balance. Cell 145 (4), 596–606. 10.1016/j.cell.2011.04.013 21565616 PMC3129781

[B227] Waqas S. F. H. Hoang A. C. Lin Y.-T. Ampem G. Azegrouz H. Balogh L. (2017). Neuropeptide FF Increases M2 Activation and Self-Renewal of Adipose Tissue Macrophages. J. Clin. Invest. 127 (7), 2842–2854. 10.1172/jci90152 28581443 PMC5490745

[B228] Watts J. L. (2009). Fat Synthesis and Adiposity Regulation in *Caenorhabditis elegans* . Trends Endocrinol. Metab. 20, 58–65. 10.1016/j.tem.2008.11.002 19181539 PMC2665873

[B229] Weaver L. N. Drummond-Barbosa D. (2019). The Nuclear Receptor Seven up Functions in Adipocytes and Oenocytes to Control Distinct Steps of Drosophila Oogenesis. Dev. Biol. 456 (2), 179–189. 10.1016/j.ydbio.2019.08.015 31470019 PMC6884690

[B230] Weaver L. N. Drummond-Barbosa D. (2020). The Nuclear Receptor Seven up Regulates Genes Involved in Immunity and Xenobiotic Response in the Adult drosophila Female Fat Body. G3 Genes, Genomes, Genet. 10 (12), 4625–4635. 10.1534/g3.120.401745 PMC7718730 33087412

[B231] Werthebach M. Stewart F. A. Gahlen A. Mettler-Altmann T. Akhtar I. Maas-Enriquez K. (2019). Control of Drosophila Growth and Survival by the Lipid Droplet-Associated Protein CG9186/Sturkopf. Cel Rep 26 (13), 3726–3740. 10.1016/j.celrep.2019.02.110 30917324

[B232] Whittle A. J. López M. Vidal-Puig A. (2011). Using Brown Adipose Tissue to Treat Obesity - the central Issue. Trends Mol. Med. 17, 405–411. 10.1016/j.molmed.2011.04.001 21602104

[B233] Wilfling F. Wang H. Haas J. T. Krahmer N. Gould T. J. Uchida A. (2013). Triacylglycerol Synthesis Enzymes Mediate Lipid Droplet Growth by Relocalizing from the ER to Lipid Droplets. Dev. Cel 24 (4), 384–399. 10.1016/j.devcel.2013.01.013 PMC3727400 23415954

[B234] Wilson E. O. (2013). Letter to a Young Scientist. 18th ed. Harvard: Liveright.

[B235] Wu J. Boström P. Sparks L. M. Ye L. Choi J. H. Giang A.-H. (2012). Beige Adipocytes Are a Distinct Type of Thermogenic Fat Cell in Mouse and Human. Cell 150 (2), 366–376. 10.1016/j.cell.2012.05.016 22796012 PMC3402601

[B236] Wu J. Cohen P. Spiegelman B. M. (2013). Adaptive Thermogenesis in Adipocytes: Is Beige the New Brown? Genes Dev. 27, 234–250. 10.1101/gad.211649.112 23388824 PMC3576510

[B237] Yadav A. Kataria M. A. Saini V. Yadav A. (2013). Role of Leptin and Adiponectin in Insulin Resistance. Clinica Chim. Acta 417, 80–84. 10.1016/j.cca.2012.12.007 23266767

[B238] Yamada T. Habara O. Kubo H. Nishimura T. (2018). Fat Body Glycogen Serves as a Metabolic Safeguard for the Maintenance of Sugar Levels in drosophila. Dev 145 (6), 65. 10.1242/dev.158865 29467247

[B239] Yamaguchi M. Yoshida H. (2018). Drosophila as a Model Organism. Adv. Exp. Med. Biol.. 1076, 1–10. 10.1007/978-981-13-0529-0_1 29951811

[B240] Yin J. Spillman E. Cheng E. S. Short J. Chen Y. Lei J. (2021). Brain-specific Lipoprotein Receptors Interact with Astrocyte Derived Apolipoprotein and Mediate Neuron-Glia Lipid Shuttling. Nat. Commun. 12 (1), 7. 10.1038/s41467-021-22751-7 33893307 PMC8065144

[B241] Young S. G. Zechner R. (2013). Biochemistry and Pathophysiology of Intravascular and Intracellular Lipolysis. Genes Develop. Genes Dev Vol. 27, 459–484. 10.1101/gad.209296.112 PMC3605461 23475957

[B242] Yu H. Dilbaz S. Coßmann J. Hoang A. C. Diedrich V. Herwig A. (2019). Breast Milk Alkylglycerols Sustain Beige Adipocytes through Adipose Tissue Macrophages. J. Clin. Invest. 129 (6), 2485–2499. 10.1172/jci125646 31081799 PMC6546455

[B243] Zhang L. Ip C. K. Lee I. J. Qi Y. Reed F. Karl T. (2018). Diet-induced Adaptive Thermogenesis Requires Neuropeptide FF Receptor-2 Signalling. Nat. Commun. 9 (1), 4722. 10.1038/s41467-018-06462-0 30413707 PMC6226433

[B244] Zhao X. Karpac J. (2017). Muscle Directs Diurnal Energy Homeostasis through a Myokine-dependent Hormone Module in Drosophila. Curr. Biol. 27 (13), 1941–1955.e6. 10.1016/j.cub.2017.06.004 28669758 PMC5533578

[B245] Zimmermann R. Strauss J. G. Haemmerle G. Schoiswohl G. Birner-Gruenberger R. Riederer M. (2004). Fat Mobilization in Adipose Tissue Is Promoted by Adipose Triglyceride Lipase. Science 306 (5700), 1383–1386. 10.1126/science.1100747 15550674

[B246] Zweytick D. Athenstaedt K. Daum G. (2000). Intracellular Lipid Particles of Eukaryotic Cells. Biochim. Biophys. Acta (Bba) - Rev. Biomembranes 1469, 101–120. 10.1016/s0005-2736(00)00294-7 10998572

